# Efficacy of Heat Mitigation Strategies on Core Temperature and Endurance Exercise: A Meta-Analysis

**DOI:** 10.3389/fphys.2019.00071

**Published:** 2019-02-13

**Authors:** Sharifah Badriyah Alhadad, Pearl M. S. Tan, Jason K. W. Lee

**Affiliations:** ^1^NUS Graduate School for Integrative Sciences and Engineering, National University of Singapore, Singapore, Singapore; ^2^Department of Physiology, Yong Loo Lin School of Medicine, National University of Singapore, Singapore, Singapore; ^3^Saw Swee Hock School of Public Health, National University of Singapore, Singapore, Singapore; ^4^Defence Medical & Environmental Research Institute, DSO National Laboratories, Singapore, Singapore; ^5^Department of Orthopaedic Surgery, Yong Loo Lin School of Medicine, National University of Singapore, Singapore, Singapore

**Keywords:** thermoregulation, aerobic fitness, heat acclimation, heat acclimatization, pre-exercise cooling, fluid ingestion

## Abstract

**Background:** A majority of high profile international sporting events, including the coming 2020 Tokyo Olympics, are held in warm and humid conditions. When exercising in the heat, the rapid rise of body core temperature (*T*_*c*_) often results in an impairment of exercise capacity and performance. As such, heat mitigation strategies such as aerobic fitness (AF), heat acclimation/acclimatization (HA), pre-exercise cooling (PC) and fluid ingestion (FI) can be introduced to counteract the debilitating effects of heat strain. We performed a meta-analysis to evaluate the effectiveness of these mitigation strategies using magnitude-based inferences.

**Methods:** A computer-based literature search was performed up to 24 July 2018 using the electronic databases: PubMed, SPORTDiscus and Google Scholar. After applying a set of inclusion and exclusion criteria, a total of 118 studies were selected for evaluation. Each study was assessed according to the intervention's ability to lower *T*_*c*_ before exercise, attenuate the rise of *T*_*c*_ during exercise, extend *T*_*c*_ at the end of exercise and improve endurance. Weighted averages of Hedges' *g* were calculated for each strategy.

**Results:** PC (*g* = 1.01) was most effective in lowering *T*_*c*_ before exercise, followed by HA (*g* = 0.72), AF (*g* = 0.65), and FI (*g* = 0.11). FI (*g* = 0.70) was most effective in attenuating the rate of rise of *T*_*c*_, followed by HA (*g* = 0.35), AF (*g* = −0.03) and PC (*g* = −0.46). In extending *T*_*c*_ at the end of exercise, AF (*g* = 1.11) was most influential, followed by HA (*g* = −0.28), PC (*g* = −0.29) and FI (*g* = −0.50). In combination, AF (*g* = 0.45) was most effective at favorably altering T_c_, followed by HA (*g* = 0.42), PC (*g* = 0.11) and FI (*g* = 0.09). AF (1.01) was also found to be most effective in improving endurance, followed by HA (0.19), FI (−0.16) and PC (−0.20).

**Conclusion:** AF was found to be the most effective in terms of a strategy's ability to favorably alter *T*_*c*_, followed by HA, PC and lastly, FI. Interestingly, a similar ranking was observed in improving endurance, with AF being the most effective, followed by HA, FI, and PC. Knowledge gained from this meta-analysis will be useful in allowing athletes, coaches and sport scientists to make informed decisions when employing heat mitigation strategies during competitions in hot environments.

## Introduction

Exercising in the heat often results in elevation in body core temperature (T_c_). This is the cumulative result of more heat being produced by the working muscles than heat loss to the environment coupled with hot and/or humid environmental conditions (Berggren and Hohwu Christensen, 1950; Saltin and Hermansen, 1966). Studies have shown that an accelerated increase in T_c_ could impair both exercise performance (i.e. time trial) and exercise capacity (i.e., time to exhaustion) (Galloway and Maughan, 1997; Parkin et al., 1999). In ambient temperatures of 4°, 11°, 21°, and 31°C, a compromise in endurance capacity due to thermoregulatory stress was already evident at 21°C (Galloway and Maughan, 1997). Parkin et al. (1999) found that time to exhaustion was longest when cycling in ambient temperatures of 3°C (85 min), followed by 20°C (60 min) and 40°C (30 min).

Elite athletes, however, cannot avoid competing in the heat since a majority of high-profile international sporting events are often held in warm conditions. The 2008 Summer Olympics in Beijing was held in average ambient conditions of 25°C with 81% relative humidity. Similarly, the 2010 Youth Olympic Games in Singapore had temperatures reaching 31°C with relative humidity between 80 and 90%. The upcoming 2020 Olympics held in Tokyo's hot and humid summer period could potentially expose athletes to one of the most challenging environmental conditions observed in the modern history of the Olympic Games, with temperatures upwards of 35°C and above 60% relative humidity. Therefore, athletes have to learn to adapt and perform in these unfavorable environments and whenever possible, incorporate mitigation strategies to counter the negative effects of heat strain to augment performance and health.

Exercise tolerance in the heat can be affected by multiple factors such as the attainment of a critically high T_c_ (Gonzalez-Alonso et al., 1999b), cardiovascular insufficiency (Gonzalez-Alonso and Calbet, 2003), metabolic disturbances (Febbraio et al., 1994b, 1996; Parkin et al., 1999) and reductions in central nervous system drive to skeletal muscle (Nybo and Nielsen, 2001; Todd et al., 2005). Indeed, a high T_c_ represents one of the key limiting factors to exercise tolerance in the heat. The development of hyperthermia has been associated with alterations in self-pacing strategies in exercise performance trials or earlier voluntary termination during exercise capacity trials (Nielsen et al., 1993; Gonzalez-Alonso et al., 1999a,b).

In order to optimize exercise tolerance in the heat, exercising individuals often employ strategies to alter T_c_. There are various ways in which this can be done, such as aerobic fitness (AF) (Nadel et al., 1974; Cheung and McLellan, 1998b), heat acclimation/acclimatization (HA) (Nielsen et al., 1993; Cotter et al., 1997), pre-exercise cooling (PC) (Gonzalez-Alonso et al., 1999a,b; Cotter et al., 2001) and fluid ingestion (FI) (Greenleaf and Castle, 1971; McConell et al., 1997). These strategies have shown to be effective in improving exercise tolerance in warm conditions through various processes that include alterations in heat dissipation ability, cardiovascular stability and adaptations and changes to the body's heat storage capacity.

Being able to objectively rank these heat mitigation strategies in order of their efficacy will be particularly useful for an athlete preparing to compete in the heat. This knowledge will also be beneficial for coaches, fitness trainers and backroom staff to discern when they consider heat mitigation in warm, humid conditions. With limited amount of time and resources, an evidence-based approach to quantify the efficacy of various heat mitigation strategies will allow selection of the most effective strategy to optimize performance and health and determine the priority in which these strategies should be employed. Furthermore, no comparison of the effect of different heat mitigation strategies have been presented using a meta-analysis thus far.

Therefore, the purpose of this review was to objectively evaluate the efficacy of various heat mitigation strategies using Hedges' g. Each study was analyzed in terms of the degree to which (i) T_c_ was lowered at the start of exercise; (ii) the rise of T_c_ is attenuated; (iii) T_c_ is extended at the end of exercise to safe limits (McLellan and Daanen, 2012) and (iv) endurance are improved. The weighted averages of Hedges' g (Hopkins et al., 2009) were then calculated, and the various heat mitigation strategies ranked in order of effectiveness in terms of both affecting T_c_ measurements and endurance.

## Materials and Methods

### Search Strategy

A computer-based literature search was performed using the following electronic databases: PubMed, SPORTDiscus and Google Scholar. The electronic database was searched with the following keywords: “fitness,” “training,” “heat acclimation,” “heat acclimatization,” “precooling,” “pre-cooling,” “cold water immersion,” “cold air,” “cold room,” “cold vest,” “cold jacket,” “ice vest,” “cold fluid,” “cold beverage,” “neck collar,” “neck cooling,” “ice slurry,” “ice slush,” “fluid ingestion,” “fluid intake,” “water ingestion,” “water intake,” “fluid replacement,” “rehydration,” “thermoregulation,” “core temperature,” and “heat mitigation.” Searches were systematically performed by combining the keywords and using Boolean operators “AND” and “OR” to yield the maximum outcome of relevant studies. Where applicable, we applied filters for language (English) and species (Human). In addition, a manual citation tracking of relevant studies and review articles was performed. The last day of the literature search was 24 July 2018.

### Inclusion and Exclusion Criteria

Studies were screened and included if they met the following criteria: (i) they investigated the effect of a heat mitigation strategy on T_c_ in an exercise context; (ii) they were conducted in warm or hot ambient conditions of more than 20°C; and (iii) they included a control condition or a pre-intervention and post-intervention assessment. Studies were excluded based on the following criteria: (i) they reported the use of pharmacological agents to alter T_c_ due to ethical issues and dangers involved with its use; (ii) they were review articles, abstracts, case studies and editorials; (iii) they involved combined use of different methods; and (iv) they involved children or the elderly.

### Data Extraction

The following data were extracted: participant characteristics, sample size, ambient conditions, exercise protocol, intervention method, exercise outcome and T_c_ measurements. T_c_ measurements included the type of T_c_ measure used, T_c_ at the beginning of exercise, rate of rise of T_c_ and T_c_ at the end of exercise. In studies where mean and standard deviation of T_c_ were not reported in the text, the relevant data was extracted using GetData Graph Digitiser (http://getdata-graph-digitizer.com). In the event that pertinent data were not available, the corresponding authors of the manuscripts were contacted. Studies with missing data that could not be retrieved or provided by the author were excluded from the meta-analysis.

### Data Analysis

In the event that rate of rise of T_c_ was not provided in the study, it was calculated as the difference between the T_c_ at the end of exercise and T_c_ at the beginning of exercise divided by the time taken to complete the task. When studies only reported standard errors, standard deviations were calculated by multiplying the standard error by the square root of the sample size.

Standardized mean differences (Hedges' g) and 95% confidence intervals (CIs) were also calculated for each study. This was derived using the mean T_c_ differences divided by the pooled standard deviation either between the control and intervention groups or between the pre-intervention and post-intervention states. A bias-corrected formula for Hedges' g for all studies was used to correct for positive and small sample bias (Borenstein et al., 2009). Weighted average of Hedges' g for each heat mitigation strategy was calculated and presented in a forest plot. A combined weighted average of Hedges' g values across all three phases for each strategy's effect on altering T_c_ and on endurance was also calculated, and used as the basis for ranking. The magnitude of the Hedges' *g*-values were interpreted as follows: < 0.20, trivial; 0.20–0.49, small; 0.50–0.79, moderate; and ≥0.80, large.

## Results

### Search Results

**Figure 1:**
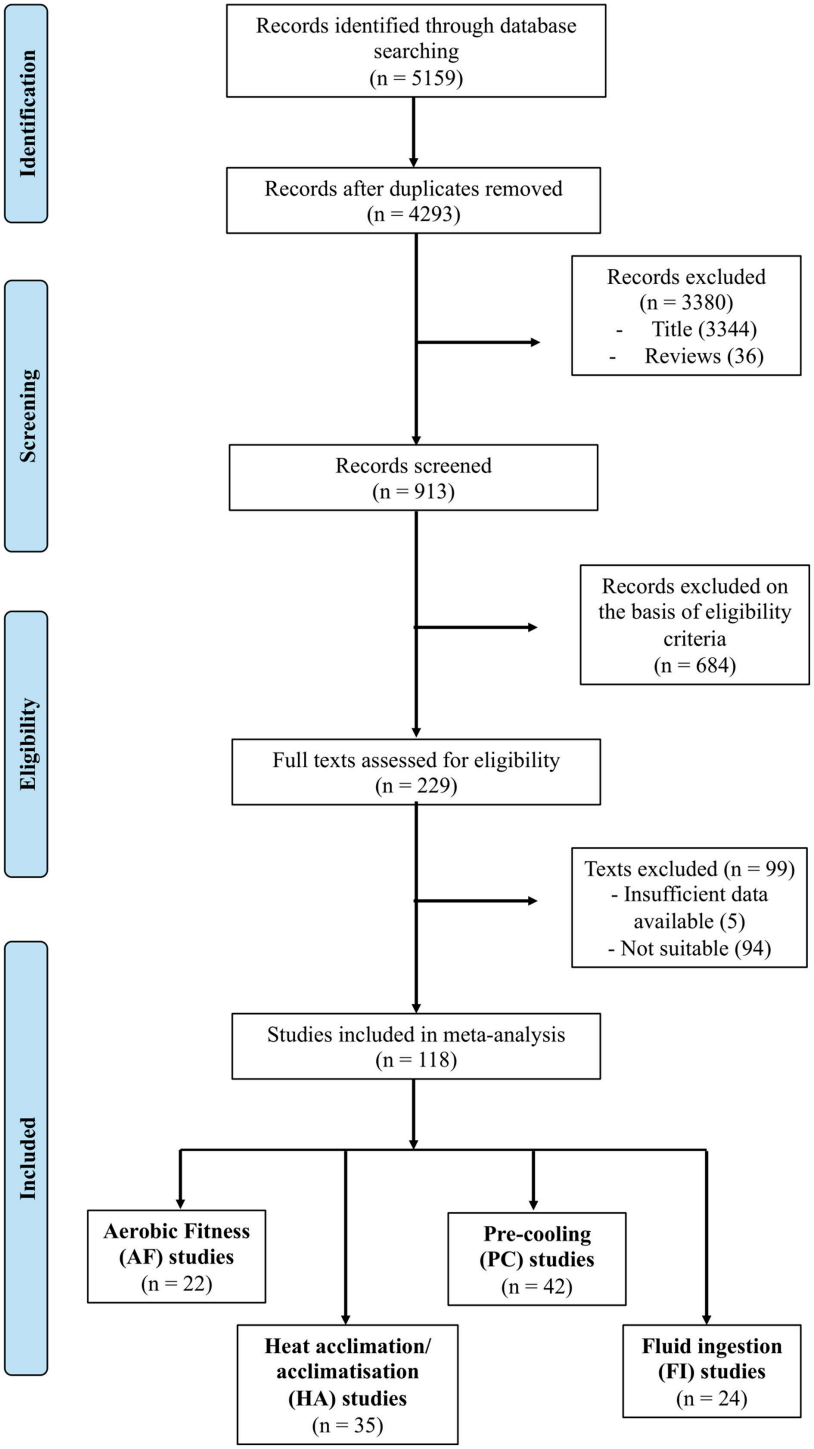
Flowchart of the study selection process.

The initial identification process yielded 5159 references and after removing duplicates and screening for title and abstract, 229 full texts were obtained. Of these, based on the assessment of study relevance and the inclusion and exclusion criteria, 118 were found to be relevant and therefore included in the analysis. The number of studies found for each heat mitigation strategy is as follows: AF (*n* = 22), HA (*n* = 35), PC (*n* = 42), and FI (*n* = 24) (Figure 1). It should be noted that AF studies may incorporate effects of HA due to the environmental conditions that the AF studies are carried out in. To separate these effects, training periods for “within subjects” AF studies included were conducted at temperatures of 30°C and below. No separation based on temperature was determined for “between subjects” studies as no training was carried out for the subjects prior to the exercise test. Characteristics of the selected studies are summarized in Tables 1–4.

**Table 1:** Summary of aerobic fitness studies.

**Study**	**Ambient conditions**	***N* =**	**Exercise protocol**	**Intervention method**	**Exercise outcome**	***T_***c***_* measure**	***T_***c***_* before**	***T_***c***_* rate of rise**	***T_***c***_* end**
Mora-Rodriguez et al., 2010	36°C 25% RH 2.5 m/s airflow	10 untrained 10 trained	EPW: Cycle at 40, 60 or 80% VO_2_ peak, equaled by total work	–	–	*T_*re*_*	Utr: 37.6 ± 0.2°C Tr: 37.4 ± 0.2°C (S)	–	–
Ichinose et al., 2005	30°C 50% RH	9	EPW: 20 min cycle at pretraining 70% VO_2_ peak under isosmotic conditions	Cycle at 60% VO_2_ peak at 30°C, 50% RH for 1 hr/day for 10 days	–	*T_*oes*_*	Before: 36.68 ± 0.15°C After: 36.53 ± 0.18°C (S)	Before: 5.31 ± 1.17°C/h After: 4.74 ± 0.97°C/h (CAL)	–
Selkirk and McLellan, 2001	40°C 30% RH < 0.1 m/s wind speed	6 untrained (low BF) 6 untrained (high BF) 6 trained (low BF) 6 trained (high BF)	EC: Treadmill walking at 3.5 km/h to exhaustion	–	Longer exercise times in Tr_low_ vs Utr_low_ and Tr_low_ vs. Tr_high_ (S)	*T_*re*_*	Utr_low_: 37.19 ± 0.20°C Tr_low_: 37.02 ± 0.20°C Utr_high_: 37.26 ± 0.37°C Tr_high_: 37.10 ± 0.22°C (NS)	Utr_low_: 1.20 ± 0.34°C/h Tr_low_: 1.27 ± 0.10°C/h Utr_high_: 1.24 ± 0.19°C/h Tr_high_: 1.55 ± 0.15°C/h (CAL)	Utr_low_: 38.58 ± 0.47°C Tr_low_: 39.48 ± 0.02°C Utr_high_: 38.78 ± 0.59°C Tr_high_: 39.22 ± 0.22°C (S)
Periard et al., 2012	40°C 50% RH 4.1 m/s convective airflow	8 untrained 8 trained	EC: Cycle to exhaustion at 60 & 75% VO_2_ max	–	No influence on times to exhaustion	*T_*re*_*	Utr_H60%_: 37.0 ± 0.3°C Tr_H60%_: 36.9 ± 0.2°C Utr_H75%_: 37.1 ± 0.3°C Tr_H75%_: 36.8 ± 0.3°C (REQ)	–	Utr_H60%_: 39.4 ± 0.4°C Tr_H60%_: 39.8 ± 0.3°C Utr_H75%_: 38.8 ± 0.5°C Tr_H75%_: 39.3 ± 0.6°C (NS)
Cheung and McLellan, 1998a	40°C 30% RH < 0.1 m/s wind speed	7 moderately fit 8 highly fit	EC: Treadmill exercise at 3.5 km/h, 0% grade in a euhydrated state to exhaustion	–	No influence on tolerance time	*T_*re*_*	MF: 36.93 ± 0.27°C HF: 36.85 ± 0.22°C (NS)	MF: 1.14 ± 0.29°C/h HF: 1.21 ± 0.27°C/h (CAL)	MF: 38.77 ± 0.27°C HF: 39.15 ± 0.18°C (NS)
Ichinose et al., 2009	25°C 45% RH	11	EPW: Cycle at 50% VO_2_ max for 30 min	Cycle at 60% VO_2_ max for 60 min/day, 4–5 days/week over 3 menstrual cycles at 30°C, 45% RH	–	*T_*oes*_*	Before: 37.27 ± 0.33°C After: 37.07 ± 0.20°C (S)	Before: 0.68 ± 0.81°C/h After: 0.80 ± 0.52°C/h (CAL)	–
Cheung and McLellan, 1998b	40°C 30% RH < 0.1 m/s wind speed	8	EC: Treadmill heat stress test in a euhydrated state to exhaustion	Treadmill walk for 1 h, 6 days/week at 60–65% VO_2_ max for 2 weeks in a normothermic environment	No influence on tolerance time	*T_*re*_*	Before: 37.08 ± 0.24°C After: 36.93 ± 0.34°C (NS)	Before: 1.04 ± 0.34°C/h After: 1.07 ± 0.30°C/h (CAL)	Before: 38.70 ± 0.37°C After: 38.61 ± 0.25°C (NS)
Wright et al., 2012	40°C 30% RH < 0.1 m/s wind speed	11 untrained 12 trained	EC: Treadmill walk at 4.5 km/h, 2% incline to exhaustion	–	Longer time to exhaustion (S)	*T_*re*_*	–	Utr: 1.25 ± 0.20°C/h Tr: 1.14 ± 0.28°C/h (NS)	Utr: 39.0 ± 0.3°C Tr: 39.7 ± 0.3°C (S)
Takeno et al., 2001	30°C 50% RH	5	EPW: 30 min cycle at 60% VO_2_ peak	Cycle at 60% VO_2_ peak for 60 min/day, 5 days/week for 2 weeks at atmospheric pressure	–	*T_*oes*_*	Before: 37.0 ± 0.2°C After: 36.8 ± 0.2°C (S)	Before: 2.6 ± 1.0°C/h After: 2.6 ± 0.6°C/h (CAL)	–
Stapleton et al., 2010	30°C 15% RH	10	EPW: 60 min cycle at a constant rate of heat production	Aerobic and resistance training for 8 weeks	–	*T_*oes*_*	Before: 37.10 ± 0.28°C After: 36.95 ± 0.24°C (S)	Before: 0.68 ± 1.8°C/h After: 0.56 ± 0.16°C/h (S)	–
Lim et al., 2009	35°C 40% RH	9 normal training 9 increased training	EC: Treadmill run at 70% VO_2_ max to exhaustion	NT: Routine training program for 14 daysIT: 20% increase in training load for 14 days	–	*T_*gi*_*	Before_NT_: 36.68 ± 0.32°C After_NT_: 36.70 ± 0.41°C Before_IT_: 36.98 ± 0.46°C After_IT_: 37.11 ± 0.39°C (NS)	Before_NT_: 3.48 ± 0.96°C/h After_NT_: 2.88 ± 1.14°C/h Before_IT_: 3.42 ± 1.20°C/h After_IT_: 3.48 ± 1.26°C/h (CAL)	–
Ho et al., 1997	36°C 20% RH	6 young sedentary 6 young fit	EPW: 20 min cycle at 35% VO_2_ peak	–	–	*T_*oes*_*	Sedentary: 37.1 ± 0.2°C Fit: 36.9 ± 0.2°C (NS)	–	–
Shvartz et al., 1977	23°C dry bulb 16°C wet bulb < 0.2 m/s wind speed	7 untrained 7 trained	EPW: 60 min bench stepping at 41 W	–	–	*T_*re*_*	Utr: 36.9 ± 0.19°C Tr: 37.1 ± 0.31°C (NS)	Utr: 1.0 ± 0.37°C/h Tr: 1.0 ± 0.42°C/h (CAL)	–
Cramer et al., 2012	24.5°C 0.9 kPa RH 1.3 m/s air velocity	10 unfit 11 fit	EPW: 60 min cycle at 60% VO_2_ max or to produce metabolic heat of 275 W/m^2^	–	–	*T_*re*_*	Unfit_60%_: 37.40 ± 0.22°C Fit_60%_: 37.09 ± 0.20°C Unfit_BAL_: 37.43 ± 0.25°C Fit_BAL_: 37.14 ± 0.23°C(S)	Unfit_BAL_: 0.93 ± 0.40°C/h Fit_BAL_: 0.95 ± 0.33°C/h (CAL)	-
Shvartz et al., 1974	21.5°C dry bulb 17.5°C wet bulb	5	EPW: 60 min bench-stepping at 85% VO_2_ max	Bench-stepping for 60 min/day for 12 days	–	*T_*re*_*	Before: 37.4 ± 0.3°C After: 37.2 ± 0.2°C (S)	–	–
Ikegawa et al., 2011	30°C 50% RH	7	EPW: 30 min cycle at 65% VO_2_ peak in a euhydrated state	Cycle for 30 min/day for 5 days	–	*T_*oes*_*	Before: 36.74 ± 0.32°C After: 36.50 ± 0.16°C (S)	Before: 3.18 ± 0.83°C/h After: 3.06 ± 0.49°C/h (CAL)	–
Yamauchi et al., 1997	23°C 60% RH	5 untrained 6 trained	EPW: 30 min cycle at 80 W	–	–	*T_*tym*_*	Utr: 36.71 ± 0.22°C Tr: 36.50 ± 0.15°C (NS)	–	–
Yamazaki et al., 1994	25°C 35% RH	8 untrained 9 trained	EPW: 30 min cycle at 35% VO_2_ max	–	–	*T_*oes*_*	Utr: 37.06 ± 0.30°C Tr: 37.02 ± 0.23°C (NS)	–	–
Gagnon et al., 2012	42°C 20% RH 1 m/s air speed	8 untrained 8 trained	EPW: 120 min cycle at 120 W with fluid replacement	–	–	*T_*oes*_*	Utr: 36.96 ± 0.25°C Tr: 36.69 ± 0.25°C (NS)	Utr: 0.68 ± 0.30°C/h Tr: 0.82 ± 0.34°C/h (CAL)	–
Merry et al., 2010	24.3°C 50% RH 4.5 m/s wind velocity	6 untrained 6 trained	EPW: 40 min cycle at 70% VO_2_ peak in a euhydrated state	–	–	*T_*rec*_*	Utr: 36.88 ± 0.26°C Tr: 36.56 ± 0.29°C (REQ)	–	–
Shields et al., 2004	32°C 32% RH	7	EPW: 45 min cycling at 40% VO_2_ peak	Exercise at 50% VO_2_ reserve for 40 min/day for 3 days per week, over 12 weeks	–	*T_*oes*_*	Before: 37.00 ± 0.27°C After: 36.88 ± 0.25°C (REQ)	Before: 0.69 ± 0.65°C/h After: 0.64 ± 0.89°C/h (REQ)	–
Smoljanic et al., 2014	25°C 37% RH	7 fit 7 unfit	EPW: Run for 60 min at 60% VO_2max_, followed by run at fixed metabolic heat production of 640 W	–	–	*T_*re*_*	–	Fit_60minrun_: 1.23 ± 0.37°C/h Unfit_60minrun_: 0.90 ± 0.30°C/h (S) Fit_fixedmetheatprod_: 0.86 ± 0.26°C/h Unfit_fixedmetheatprod:_ 0.92 ± 0.32°C/h (NS)	–

*RH, relative humidity; EC, exercise capacity; EP, exercise performance; EPW, exercise performance at a fixed workload; S, significant; NS, not significant; CAL, calculated values; REQ, requested values; T_re_, rectal temperature; T_oes_, oesophageal temperature; T_gi_, gastrointestinal temperature; T_tym_, tympanic temperature; Utr, untrained subjects; Tr, trained subjects; BF, body fat; NT, normal training; IT, increased training; MF, moderately fit subjects; HF, highly fit subjects; AF, aerobic fitness*.

**Table 2:** Summary of heat acclimation/acclimatization studies.

**Study**	**Ambient conditions**	***N* =**	**Exercise protocol**	**Intervention method**	**Exercise outcome**	***T_***c***_* measure**	***T_***c***_* before**	***T_***c***_* rate of rise**	***T_***c***_* end**
**LOW HUMIDITY (<50% RH)**									
Lorenzo and Minson, 2010	38°C 30% RH	12	EP: 1 h cycling time trial	Two 45 min exposures to 40°C, 30% RH conditions for 10 days	Higher power output (S)	*T_*re*_*	Before: 37.1 ± 0.3°C After: 37.0 ± 0.4°C (REQ)	–	Before: 39.5 ± 0.3°C After: 39.4 ± 0.7°C (NS)
Cheung and McLellan, 1998a	40°C 30% RH < 0.1 m/s wind speed	7 moderately fit 8 highly fit	EC: Treadmill walk at 3.5 km/h, 0% grade in a euhydrated state to exhaustion	1 h exposures to 40°C, 30% RH conditions for 5 days/week for 2 weeks	No influence on tolerance time	*T_*re*_*	Before_MF_: 36.93 ± 0.27°C After_MF_: 36.96 ± 0.28°C Before_HF_: 36.85 ± 0.22°C After_HF_: 36.74 ± 0.19°C (NS)	Before_MF_: 1.14 ± 0.29°C/h After_MF_: 1.08 ± 0.25°C/h Before_HF_: 1.21 ± 0.27°C/h After_HF_: 1.25 ± 0.20°C/h (CAL)	Before_MF_: 38.77 ±0.27°C After_MF_: 38.79 ± 0.31°C Before_HF_: 39.15 ± 0.18°C After_HF_: 39.14 ± 0.21°C (NS)
Nielsen et al., 1993	40°C 10% RH	8	EC: Cycling at approximately 50% VO_2_ max to exhaustion	90 min exposures to 40°C, 10% RH conditions for 9–12 days	Increase in endurance time (S)	*T_*oes*_*	–	–	Before: 39.8 ± 0.4°C After: 39.7 ± 0.4°C (NS)
Horstman and Christensen, 1982	45°C dry bulb 23°C wet bulb	6 men 4 women	EPW: 120 min cycle at 40% VO_2_ max	2 h exposures to 45°C dry bulb, 23°C wet bulb conditions for 11 days	–	*T_*re*_*	–	Before_men_: 1.5 ± 0.5°C/h After_men_: 0.8 ± 0.2°C/h (NS) Before_women_: 1.4 ± 0.4°C/h After_women_: 0.5 ± 0.0°C/h (S)	–
Weller et al., 2007	46.1°C dry bulb 17.9% RH	8 in RA_12_ 8 in RA_26_	EPW: 60 min treadmill walk at 45% VO_2_ peak	100 min exposures to 46.1°C, 17.9% RH conditions for 10 days	–	*T_*re*_*	Before_12_: 37.20 ± 0.27°C After_12_: 36.95 ± 0.22°C Before_26_: 37.27 ± 0.15°C After_12_: 37.00 ± 0.13°C (S)	Before_12_: 1.39 ± 0.41°C/h After_12_: 1.17 ± 0.37°C/h Before_26_: 1.42 ± 0.28°C/h After_12_: 1.16 ± 0.21°C/h (CAL)	–
Shvartz et al., 1977	23°C dry bulb16°C wet bulb < 0.2 m/s wind speed	7 untrained 7 trained	EPW: 60 min bench stepping at 41 W	3 h exposures to 39.4°C dry bulb, 30.3°C wet bulb conditions for 8 days	–	*T_*re*_*	Before_Utr_: 37.1 ± 0.31°C After_Utr_: 36.7 ± 0.20°C Before_Tr_: 36.9 ± 0.19°C After_Tr_: 36.7 ± 0.13°C (S)	Before_Utr_: 1.0 ± 0.37°C/h After_Utr_: 1.0 ± 0.27°C/h Before_Tr_: 1.0 ± 0.42°C/h After_Tr_: 0.9 ± 0.21°C/h (CAL)	–
Febbraio et al., 1994a	40°C 20% RH	13	EPW: 40 min cycle at 70% VO_2_ max	90 min exposures to 40°C, 20% RH conditions for 7 days	–	*T_*re*_*	Before: 37.2 ± 0.4°C After: 36.8 ± 0.4°C (NS)	Before: 3.8 ± 0.8°C/h After: 3.6 ± 0.8°C/h (CAL)	–
Beaudin et al., 2009	24°C 30% RH	8	EC: Incremental cycling to exhaustion	2 h passive exposures to 50°C, 20% RH conditions for 10 days	–	*T_*oes*_*	Before: 37.57 ± 0.23°C After: 37.32 ± 0.14°C (S)	–	–
Magalhaes Fde et al., 2006	40°C 32% RH	6	EPW: 60 min cycle at 50% VO_2_ peak	1 h exposures to 40°C, 32% RH conditions for 9 days	–	*T_*re*_*	Before: 37.2 ± 0.2°C After: 37.0 ± 0.2°C (S)	Before: 0.94 ± 0.16°C/h After: 0.88 ± 0.27°C/h (NS)	–
Armstrong et al., 1985	40.1°C 23.5% RH	9	EPW: 90 min treadmill walk at 5.6 km/h, 6% grade with a high or low sodium diet	90 min exposures to 40.1°C, 23.4% RH conditions for 8 days	–	*T_*re*_*	Before_low_: 37.44 ± 0.66°C After_low_: 37.05 ± 0.30°C (S) Before_high_: 37.25 ± 0.72°C After_high_: 36.97 ± 0.45°C (NS)	Before_low_: 0.85 ± 0.48°C/h After_low_: 0.74 ± 0.26°C/h Before_high_: 0.93 ± 0.59°C/h After_high_: 0.79 ± 0.36°C/h (CAL)	–
Watkins et al., 2008	39.5°C 27% RH	10	EPW: 30 min cycle at 75% VO_2_ peak	30 min exposures to 39.5°C27% RH conditions for 7 days	–	*T_*re*_*	Before: 37.2 ± 0.2°C After: 37.0 ± 0.2°C (S)	Before: 1.8 ± 0.9°C/h After: 1.8 ± 0.5°C/h (CAL)	–
Burk et al., 2012	42°C 18% RH	21	EC: Treadmill walk at 60% VO_2_ peak to exhaustion	Two 50 min exposures to 42°C, 18% RH conditions for 10 days	Increase in endurance time (S)	*T_*re*_*	Before: 37.2 ± 0.2°C After: 37.0 ± 0.2°C (S)	Before: 1.7 ± 0.4°C/h After: 1.0 ± 0.3°C/h (CAL)	Before: 39.7 ± 0.4°C After: 39.7 ± 0.4°C (NS)
Hodge et al., 2013	35.3°C 40.2% RH	8	EPW: 90 min treadmill walk at 40% VO_2_ max	90 min exposures to 35.3°C, 40.2% RH conditions for 8 days	–	*T_*re*_*	Before: 37.1 ± 0.3°C After: 36.8 ± 0.4°C(REQ)	Before: 1.8 ± 0.3°C/h After: 0.7 ± 0.4°C/h (REQ)	–
Magalhaes Fde et al., 2010	40°C 45% RH	9	EPW: 90 min treadmill run at 50% maximal power output	90 min exposures to 40°C, 45% RH conditions for 11 days	–	*T_*re*_*	Before: 37.43 ± 0.17°C After: 37.26 ± 0.18°C (REQ)	Before: 1.05 ± 0.29°C/h After: 1.03 ± 0.23°C/h (REQ)	–
Racinais et al., 2012	44°C 44% RH	18	EPW: 30 min treadmill walk at 5 km/h, 1% grade	Football training in 38–43°C, 12–30% RH conditions for 6 days	–	*T_*re*_*	Before: 37.37 ± 0.17°C After: 37.26 ± 0.23°C (REQ)	Before: 1.18 ± 0.51°C/h After: 1.24 ± 0.62°C/h (REQ)	–
Best et al., 2014	35°C 40% RH	7	EPW: 60 min cycle at 70% VO_2max_	60 min cycling at 70% VO_2max_ in 35°Cm, 40% conditions for 6 days	–	*T_*re*_*	–	–	Before: 39.1 ± 0.3°C After: 38.7 ± 0.3°C (S) (Graph)
Dileo et al., 2016	45°C 20% RH	10	EC: Ramped running protocol until volitional fatigue	2 × 45 min periods cycling at 50% VO_2max_ in 45°C, 20% RH conditions for 5 days	–	*T_*re*_*	Before: 36.9 ± 0.2°C After: 36.7 ± 0.2°C (NS) (Graph)	–	Before: 38.9 ± 0.6°C After: 38.7 ± 0.4°C (S)
Flouris et al., 2014	40°C 20% RH	10	EPW: Cycle at fixed rates of metabolic heat production equal to 300, 350 and 400 W/m^2^, for 30 min each	90 min cycling at 50% VO_2peak_ in 40°C, 20% RH for 14 days	–	*T_*re*_*	Before: 37.0 ± 0.2 °C After: 36.7 ± 0.1°C (S) (Graph)	–	–
Gibson et al., 2015	40°C 28% RH	24	EPW: 30 min running at 9 km/h and 2% elevation	FIXED protocol: 90 min of cycling at 50% VO_2peak_ in 40°C, 39% RH ISO_CONT_: Cycle at 65% VO_2peak_ until Tre of 38.5°C reached ISO_PROG_: Cycle at 65% VO2_peak_ until Tre of 38.5°C reached for first 5 days, (then until 39°C for last 5 days). STHA – Protocol above for 5 days LTHA – Protocol above for 10 days	–	*T_*re*_*	Before (FIXED): 37.2 ± 0.4°C Before (ISO_CONT_): 37.1 ± 0.2°C Before (ISO_PROG_): 36.9 ± 0.4°C STHA - Before (FIXED): 36.9 ± 0.4°C Before (ISO_CONT_): 37.0 ± 0.2°C Bssefore (ISO_PROG_): 36.7 ± 0.4°C (S) LTHA - Before (FIXED): 36.9 ± 0.4°C Before (ISO_CONT_): 37.0 ± 0.2°C Before (ISO_PROG_): 36.8 ± 0.3°C (S)	Before (FIXED): 2.35 ± 0.87°C/h Before (ISO_CONT_): 3.21 ± 0.6°C/h Before (ISO_PROG_): 2.97 ± 0.4°C/h STHA – After (FIXED): 2.49 ± 1.13°C/h After (ISO_CONT_): 2.77 ± 0.71°C/h After (ISO_PROG_): 2.87 ± 0.49°C/h LTHA - After (FIXED): 2.39 ± 0.94°C/h After (ISO_CONT_): 2.56 ± 0.75°C/h After (ISO_PROG_): 2.82 ± 0.78°C/h	–
Racinais et al., 2015b	34°C 18% RH	9	EP: 43.3 km cycling time trial	4 h exposures to 34°C, 18% RH conditions for 2 weeks	Faster time trial (S)	*T_*re*_*	–	–	Before: 40.2 ± 0.4°C After: 40.1 ± 0.4°C
**HIGH HUMIDITY (>** **50% RH)**									
Cotter et al., 1997	39.5°C 59.2% RH	8	EPW: 70 min cycle at 50% peak aerobic power	70 min exposures to 39.5°C, 59.2% RH conditions for 6 days	–	*T_*ac*_*	Before: 36.83 ± 0.05°C After: 36.62 ± 0.05°C (S)	–	–
Fujii et al., 2012	37°C 50% RH < 0.2 m/s wind speed	10	EPW: 75 min cycle at 58% VO_2_ peak	Four 20 min exposures to 37°C conditions for 6 days	–	*T_*oes*_*	Before: 36.6 ± 0.1°C After: 36.4 ± 0.2°C (S)	–	–
Buono et al., 1998	35°C 75% RH	9	EPW: 2 h exercise bouts of either a treadmill walk at 1.34 m/s, 3% grade or a cycle at 75 W	Either treadmill walking at 1.34 m/s, 3% grade or cycling at 75 W in 35°C, 75% RH conditions	–	*T_*re*_*	Before: 37.0 ± 0.3°C After: 36.7 ± 0.4°C (S)	Before: 1.0 ± 0.2°C/h After: 0.8 ± 0.3°C/h (CAL)	–
Lee et al., 2012	32°C dry bulb 70% RH 400 W/m^2^ solar radiation	18	EPW: Three 60 min marches on the treadmill at 4 km/h, 0% gradient in Skeletal Battle Order (SBO) or Full Battle Order (FBO)	Outdoor route marches at 4 km/h in 29°C, 80% RH conditions for 10 days	–	*T_*gi*_*	Before_SBO_: 37.2 ± 0.3°C After_SBO_: 37.0 ± 0.3°C Before_FBO_: 37.1 ± 0.4°C After_FBO_: 37.0 ± 0.3°C (NS)	Before_SBO_: 0.4 ± 0.2°C/h After_SBO_: 0.4 ± 0.2°C/h Before_FBO_: 0.4 ± 0.2°C/h After_FBO_: 0.5 ± 0.2°C/h (CAL)	–
Kotze et al., 1977	32.2°C wet bulb 33.9°C dry bulb 0.4 m/s wind velocity	4	EPW: 4 h block stepping at an external workload after receiving placebo	4 h exposures to 32.2°C wet bulb, 33.9°C dry bulb conditions for 10 days	–	*T_*re*_*	Before: 37.5 ± 0.2°C After: 37.1 ± 0.2°C	Before: 0.5 ± 0.1°C/h After: 0.3 ± 0.1°C/h (CAL)	–
Kobayashi et al., 1980	33.5°C 60% RH	5	EPW: 60 min cycle at 60 to 70% VO_2_ max	100 min exposures to 45 to 50°C, 30 to 40% RH conditions for 9 days	–	*T_*re*_*	Before: 37.4 ± 0.2°C After: 37.0 ± 0.4°C (S)	Before: 2.0 ± 0.4°C/h After: 2.2 ± 0.5°C/h (CAL)	–
Saat et al., 2005	31.1°C 70% RH	16	EPW: 60 min cycle at 60% VO_2_ max	60 min exposures to 31.1°C, 70% RH conditions for 14 days	–	*T_*re*_*	Before: 37.35 ± 0.34°C After: 37.14 ± 0.32°C (NS)	–	–
Patterson et al., 2004	39.8°C 59.2% RH	6	EPW: 90 min cycle at ~44% W_peak_	90 min exposures to 40°C, 60% RH conditions for 16 days	–	*T_*oes*_*	Before: 36.97 ± 0.20°C After: 36.74 ± 0.14°C (REQ)	Before: 1.27± 0.15°C/h After: 1.04± 0.31°C/h (REQ)	–
Garrett et al., 2009	35°C 60% RH	10	EPW: 90 min cycling at 40% peak power output	90 min exposures to 40°C, 60% RH conditions for 5 days	–	*T_*re*_*	Before: 37.05 ± 0.37°C After: 36.95 ± 0.26°C (REQ)	Before: 1.03± 0.41°C/h After: 0.90± 0.31°C/h (REQ)	–
Garrett et al., 2012	35°C 60% RH	8	EPW: 10 min rowing at 30% peak power output, followed by 10 min rowing at 60% peak power output	90 min exposures to 39.5°C, 60% RH conditions for 5 days	–	*T_*re*_*	Before: 37.33 ± 0.16°C After: 37.28 ± 0.28°C (REQ)	Before: 2.04± 0.82°C/h After: 1.38± 0.98°C/h (REQ)	–
James C. A. et al., 2017	32°C 60% RH	10	EP: 5 km running time trial	90 min exposures to 37°C, 59% RH conditions for 5 days	Faster time trial time (S)	*Tre*	Before: 36.97 ± 0.33°C After: 36.83 ± 0.32°C (S)	–	
James et al., 2018	32°C 60% RH	9	EP: 5 km running time trial	90 min exposures to 37°C, 60% RH conditions for 5 days	Faster time trial time (S)	*T_*re*_*	Before: 37.12 ± 0.22°C After: 37.03 ± 0.23°C (NS)	Before: 5.41 ± 0.91°C/h After: 5.56 ± 0.25°C/h (CAL)	Before: 39.34 ± 0.3°C After: 39.16 ± 0.44°C (S)
Willmott et al., 2016	30°C 60% RH	14	EP: 5 km running time trial	STHA: 45 min cycling at 50% VO_2peak_ at 35°C, 60% RH once for 4 daysTDHA: 45 min cycling at 50% VO_2peak_ at 35°C, 60% twice daily for 2 days	No influence on time trial time.	*T_*re*_*	STHA - Before: 37.5 ± 0.4°C After: 37.3 ± 0.3°C (NS) TDHA – Before: 37.4 ± 0.3°C After: 37.3 ± 0.2°C (NS) (Graph)		STHA - Before: 38.69 ± 0.38°C After: 38.53 ± 0.45°C (NS) TDHA – Before: 38.59 ± 0.37°C After: 38.52 ± 0.5°C (NS) (Graph)
Brade et al., 2013	35°C 60% RH	10	EPW: 70 min repeat sprint protocol	32–48 min cycling exposure at 35°C, 60% RH conditions for 5 days	No influence on performance	*T_*gi*_*	Before: 37.0 ± 0.4°C After: 36.9 ± 0.3°C	Before: 1.54 ± 0.48°C/h After: 1.37 ± 0.36°C/h (CAL)	Before: 38.8 ± 0.4°C After: 38.5 ± 0.3°C
Zimmermann et al., 2018	35°C 50% RH	8	EP: 800 kJ cycling time trial	60 min cycling at 50% VO_2peak_ at 35°C, 49% RH conditions for 10 days (5 days on, 2 off, 5 days on)	Faster cycling time	*T_*gi*_*	Before: 36.9 ± 0.3°C After: 36.7 ± 0.4°C	Before: 3.23 ± 1.31 °C/h After: 3.57 ± 1.04°C/h	Before: 39.0 ± 0.8°C After: 38.9 ± 0.5°C

*RH, relative humidity; EC, exercise capacity; EP, exercise performance; EPW, exercise performance at a fixed workload; S, significant; NS, not significant; CAL, calculated values; REQ, requested values; Graph, graph-extracted values; T_re_, rectal temperature; T_oes_, oesophageal temperature; T_ac_, auditory canal temperature; T_gi_, gastrointestinal temperature; MF, moderately fit subjects; HF, highly fit subjects; STHA, Short term Heat acclimation and acclimatization (HA); TDHA, Twice daily HA; LTHA, Long term HA*.

**Table 3:** Summary of pre-event cooling studies.

**Study**	**Ambient conditions**	***N* =**	**Exercise protocol**	**Intervention method**	**Exercise outcome**	***T_***c***_* measure**	***T_***c***_* before**	***T_***c***_* rate of rise**	***T_***c***_* end**
**COLD WATER IMMERSION**									
Kay et al., 1999	31.4°C 60.2% RH	7	EP: 30 min self-paced cycling time trial	CON: 30 min rest INT: Whole body water immersion for 58.6 min	Greater distance covered (S)	*T_*re*_*	–	–	CON: 38.7 ± 0.3°C INT: 38.4 ± 0.5°C (NS)
Booth et al., 1997	32°C 60% RH	8	EP: 30 min running time trial	CON: No cooling INT: Cold water immersion for 60 min before exercise	Greater distance covered (S)	*T_*re*_*	CON: 37.4 ± 1.1°C INT: 36.7 ± 0.3°C (S)	–	CON: 39.6 ± 0.6°C INT: 38.9 ± 0.6°C (NS)
Tsuji et al., 2012	37°C 50% RH	10	EC: Cycle at 50% VO_2_ peak to exhaustion	CON: 25 min immersion in 35°C water INT: 25 min immersion in 18°C water	Longer time to exhaustion (S)	*T_*oes*_*	CON: 36.9 ± 0.3°C INT: 36.1 ± 0.3°C (S)	–	–
Gonzalez-Alonso et al., 1999b	40°C 19% RH	7	EC: Cycle at 60% VO_2_ max to exhaustion	CON: 30 min immersion in 36°C water INT: 30 min immersion in 17°C water	Longer time to exhaustion (S)	*T_*oes*_*	CON: 37.4 ± 0.3°C INT: 35.9 ± 0.5°C (S)	CON: 3.7 ± 0.1°C/h INT: 4.0 ± 0.1°C/h (CAL)	CON: 40.2 ± 0.3°C INT: 40.1 ± 0.3°C (NS)
Yeargin et al., 2006	27°C	15	EP: 2 mile time trial	CON: No cooling (mock treatment)INT: 12 min immersion in 14°C water during recovery	Shorter run time (S)	*T_*re*_*	CON: 37.82 ± 0.54°C INT: 37.39 ± 0.77°C (S)	–	CON: 38.87 ± 0.50°C INT: 38.59 ± 0.58°C (S)
Barr et al., 2011	49°C 12% RH	8	EPW: 20 min treadmill walk at 5 km/h, 7.5% grade	CON: No coolingINT: 15 min hand/forearm immersion during recovery	–	*T_*gi*_*	CON: 38.3 ± 0.2°C INT: 38.0 ± 0.2°C (S)	CON: 2.7 ± 0.8°C/hINT: 2.4 ± 1.1°C/h(CAL)	–
Wilson et al., 2002	21.3°C 22.4% RH	8	EPW: 60 min cycle at 60% VO_2_ max	CON: 30 min immersion in 35°C waterINT: 30 min immersion in 18°C water	–	*T_*re*_*	CON: 36.81 ± 0.25°C INT: 36.14 ± 0.51°C (S)	–	–
Smith et al., 2013	21.6°C 20% RH	10	EC: Incremental treadmill protocol beginning at 2.7 km/h, 10% grade	CON: No cooling INT: 24 min immersion in 23°C water	Shorter time to exhaustion (S)	*T_*gi*_*	CON: 37.1 ± 0.4°C INT: 36.6 ± 0.3°C (S)	CON: 2.0 ± 1.1°C/h INT: 1.2 ± 1.4°C/h (CAL)	CON: 37.6 ± 0.4°C INT: 36.9 ± 0.3°C (S)
Duffield et al., 2010	33°C 50% RH	8	EP: 40 min cycling time trial	CON: No cooling INT: 20 min lower body immersion in 14°C water	Greater mean power (S)	*T_*re*_*	CON: 37.6 ± 0.3°C INT: 37.7 ± 0.3°C (REQ)	–	CON: 39.0 ± 0.4°C INT: 38.9 ± 0.3°C (REQ)
Siegel et al., 2012	34.0°C 52% RH	8	EC: Treadmill run at first ventilatory threshold to exhaustion	CON: No cooling INT: 30 min immersion in 24°C water	Longer time to exhaustion (S)	*T_*re*_*	CON: 37.11 ± 0.28°C INT: 37.14 ± 0.34°C (REQ)	CON: 2.88 ± 0.96°C/h INT: 2.28 ± 1.56°C/h (CAL)	CON: 39.48 ± 0.36°C INT: 39.48 ± 0.34°C (NS)
Hasegawa et al., 2006	32°C 80% RH	9	EPW: 60 min cycle at 60% VO_2_ max	CON: No cooling INT: 30 min immersion in 25°C water	–	*T_*re*_*	CON: 37.36 ± 0.15°C INT: 36.80 ± 0.30°C (REQ)	CON: 1.76 ± 0.21°C/h INT: 1.85 ± 0.48°C/h (REQ)	–
Castle et al., 2006	34°C 52% RH	12	EPW: 40 min intermittent cycling sprint protocol	CON: No cooling INT: 20 min immersion in 18°C water	More work done (S)	*T_*re*_*	CON: 37.5 ± 0.1°C INT: 37.1 ± 0.1°C (S) (Graph)	CON: 2.3 ± 0.3°C/h INT: 2.0 ± 0.4°C/h (CAL)	CON: 39.0 ± 0.1°C INT: 38.4 ± 0.1°C (S) (Graph)
Clarke et al., 2017	32°C 47% RH	8	EPW: 90 min treadmill run at 65% VO_2_ max	CON: 60 min rest INT: 60 min immersion in 20°C water	–	*T_*re*_*	CON: 36.7 ± 0.3°C INT: 35.7 ± 0.9°C (S) (Graph)	CON: 1.5 ± 0.3°C/h INT: 2.1 ±°C/h (CAL)	CON: 38.9 ± 0.5°C INT: 38.8 ± 0.5°C (NS) (Graph)
Lee et al., 2018	32°C 47% RH	8	EPW: 90 min treadmill run at 65% VO_2_max	CON: 60 min rest INT: 60 min immersion in 20°C water	–	*T_*re*_*	CON: 36.7 ± 0.3°C INT: 35.7 ± 0.9°C (S) (Graph)	CON: 1.56 ± 0.45°C/h INT: 2.15 ± 0.72°C/h (S)	CON: 38.9 ± 0.5°C INT: 38.9 ± 0.5°C
Skein et al., 2012	31°C 33% RH	10	EPW: 50 min self-paced intermittent sprint exercise protocol	CON: 15 min rest INT: 15 min immersion in 10°C water	Longer total sprint time (S)	*T_*gi*_*	CON: 37.3 ± 0.2°C INT: 36.8 ± 0.4°C (S) (Graph)	–	CON: 38.9 ± 0.5°C INT: 38.7 ± 0.7°C (NS) (Graph)
Stevens et al., 2017	33°C 46% RH	9	EP: 5 km self-paced running time trial	CON: No cooling INT: 30 min immersion in 23–24°C water	Faster running time (S)	*T_*re*_*	CON: 37.3 ± 0.3°C INT: 36.7 ± 0.4°C (S) (Graph)	CON: 3.8 ± 0.3°C/h INT: 4.7 ± 0.3°C/h (CAL)	CON: 38.9 ± 0.3°C INT: 38.6 ± 0.4°C (S) (Graph)
**COLD AIR EXPOSURE**									
Lee and Haymes, 1995	24°C 51–52% RH	14	EC: Treadmill run at 82% VO_2_ max to exhaustion	CON: 30 min rest in a 24°C, 53% RH room INT: 33 min rest in a 5°C, 68% RH room	Longer time to exhaustion (S)	*T_*re*_*	–	CON: 3.86 ± 0.51°C/h INT: 3.76 ± 0.54°C/h (CAL)	CON: 38.02 ± 0.46°C INT: 37.86 ± 0.53°C (NS)
Olschewski and Bruck, 1988	18°C 50% RH	6	EC: Cycling with a constant increase in workload to exhaustion	CON: No cooling INT: Double cold air exposure before starting exercise	Longer time to exhaustion (S)	*T_*oes*_*	–	–	CON: 38.94 ± 0.34°C INT: 38.64 ± 0.27°C (S)
**COLD VEST OR ICE VEST**									
Stannard et al., 2011	24–26°C 29–33% RH	8	EP: 10 km running time trial	CON: Wearing a t-shirt INT: Wearing a cooling vest for 30 min before time trial	No influence on run time	*T_*gi*_*	CON: 37.7 ± 0.72°C INT: 37.3 ± 0.73°C (NS)	–	–
Arngrimsson et al., 2004	32°C 50% RH	17	EP: 5 km running time trial	CON: Wearing a t-shirt INT: Wearing an ice vest for 38 min before time trial	Shorter run time (S)	*T_*oes*_*	CON: 37.4 ± 0.4°C INT: 37.1 ± 0.5°C (S)	–	CON: 39.8 ± 0.4°C INT: 39.7 ± 0.4°C (REQ)
Kenny et al., 2011	35°C 65% RH	10	EPW: 120 min treadmill walk at 3 miles/h, 2% grade	CON: NBC suit without ice vest INT: NBC suit with ice vest	–	*T_*oes*_*	CON: 36.88 ± 0.13°C INT: 36.94 ± 0.25°C (NS)	CON: 1.08 ± 0.22°C/h INT: 0.90 ± 0.24°C/h (CAL)	–
Bogerd et al., 2010	29.3°C 80% RH	8	EPW: 60 min cycle at 65% VO_2_ peak	CON: No coolingINT: Wearing an ice vest for 45 min before exercise	–	*T_*re*_*	CON: 37.0 ± 0.2°C INT: 37.1 ± 0.2°C (NS)	CON: 2.1 ± 0.54°C/h INT: 2.0 ± 0.54°C/h (CAL)	–
Barr et al., 2011	49°C 12% RH	8	EPW: 20 min treadmill walk at 5 km/h, 7.5% grade	CON: No cooling INT: Wearing an ice vest for 15 min during recovery	–	*T_*gi*_*	CON: 38.3 ± 0.2°C INT: 38.2 ± 0.1°C (NS)	CON: 2.7 ± 0.8°C/h INT: 2.7 ± 0.4°C/h (CAL)	-
Quod et al., 2008	34.3°C 41.2% RH	6	EP: 40 min cycling time trial	CON: No cooling INT: Wearing a cooling jacket for 40 min before exercise	No influence on cycling time	*T_*re*_*	–	–	CON: 39.6 ± 0.4°C INT: 39.7 ± 0.5°C (REQ)
Brade et al., 2014	35°C 60% RH	12	EPW: 70 min repeat sprint protocol	CON: No cooling INT: Wearing a cooling jacket for 30 min before exercise	No influence on performance	*T_*gi*_*	CON: 37.0 ± 0.4°C INT: 36.9 ± 0.3°C	CON: 1.6 ± 0.3°C/h INT: 1.7 ± 0.3°C/h (CAL)	CON: 38.9 ± 0.3°C INT: 38.9 ± 0.5°C
Castle et al., 2006	34°C 52% RH	12	EPW: 40 min intermittent cycling sprint protocol	CON: No cooling INT: Wearing an ice vest for 20 min before exercise	More work done (S)	*T_*re*_*	CON: 37.5 ± 0.1°C INT: 37.3 ± 0.1°C (NS) (Graph)	CON: 2.3 ± 0.3°C/h INT: 2.3 ± 0.5°C/h (CAL)	CON: 39.0 ± 0.1°C INT: 38.8 ± 0.2°C (NS) (Graph)
Faulkner et al., 2015	35°C 51% RH	10	EPW: 1 h cycling time trial at 75% W_max_	CON: No cooling INT_COLD_: Wearing a frozen cooling garment for 30 min before exercise INT_COOL_: Wearing a cooling garment saturated in 14°C water for 30 min before exercise	Faster time trial for COLD (S) No influence on performance for COOL	*T_*gi*_*	CON: 36.7 ± 0.4°C INT_COLD_: 36.5 ± 0.3°C INT_COOL_: 36.7 ± 0.6°C (NS)	CON: 1.9 ± 0.3°C/h INT_COLD_: 2.2 ± 0.2°C/h INT_COOL_: 1.9 ± 0.4°C/h (CAL)	CON: 38.6 ± 0.5°C INT_COLD_: 38.7 ± 0.4°C INT_COOL_: 38.6 ± 0.5°C (NS)
**COLD FLUID INGESTION**									
Byrne et al., 2011	32°C dry bulb 60% RH 3.2 m/s air velocity	7	EP: 30 min self-paced cycling time trial	CON: 37°C fluid INT: 2°C fluid	Greater distance covered (S)	*T_*re*_*	–	–	CON: 38.6 ± 0.5°C INT: 38.1 ± 0.3°C (NS)
Lee et al., 2008	35.0°C 60% RH	8	EC: Cycle at 65% VO_2_ peak to exhaustion	CON: Warm drink (37°C) INT: Cold drink (4°C)	Longer time to exhaustion (S)	*T_*re*_*	CON: 36.8 ± 0.3°C INT: 36.4 ± 0.3°C (S)	CON: 3.0 ± 0.2°C/h INT: 2.9 ± 0.2°C/h (REQ)	CON: 39.4 ± 0.4°C INT: 39.5 ± 0.4°C (REQ)
**ICE SLURRY INGESTION**									
Siegel et al., 2012	34.0°C 52% RH	8	EC: Treadmill run at first ventilatory threshold to exhaustion	CON: Warm fluid (37°C) INT: Ice slurry mixture (−1°C)	Longer time to exhaustion (S)	*T_*re*_*	CON: 37.11 ± 0.28°C INT: 36.70 ± 0.31°C (REQ)	CON: 2.88 ± 0.96°C/h INT: 3.60 ± 1.20°C/h (CAL)	CON: 39.48 ± 0.36°C INT: 39.76 ± 0.36°C (S)
Siegel et al., 2010	34.0 ± 0.2°C 54.9 ± 5.9% RH	10	EC: Treadmill run at first ventilatory threshold to exhaustion	CON: Cold water (4°C) INT: Ice slurry (−1°C)	Longer time to exhaustion (S)	*T_*re*_*	CON: 36.87 ± 0.11°C INT: 36.55 ± 0.16°C (REQ)	CON: 3.00 ± 0.72°C/h INT: 3.24 ± 0.48°C/h (CAL)	CON: 39.05 ± 0.37°C INT: 39.36 ± 0.41°C (S)
Stanley et al., 2010	34°C 60% RH	10	EP: Perform a set amount of work in as fast a time as possible	CON: Cold liquid beverage (18.4°C) INT: Ice-slush beverage (−0.8°C)	No influence on cycle time	*T_*re*_*	CON: 37.4 ± 0.2°C INT: 37.0 ± 0.3°C (S)	–	CON: 39.1 ± 0.4°C INT: 39.0 ± 0.5°C (NS)
Yeo et al., 2012	28.2°C wet bulb globe temperature	11	EP: 10 km outdoor running time trial	CON: Ambient temperature drink (30.9°C) INT: Ice slurry (-1.4°C)	Faster performance time (S)	*T_*gi*_*	CON: 37.2 ± 0.3°C INT: 36.9 ± 0.3°C (REQ)	–	CON: 39.8 ± 0.4°C INT: 40.2 ± 0.6°C (S)
Brade et al., 2014	35°C 60% RH	12	EPW: 70 min repeat sprint protocol	CON: No cooling INT: Ice slurry (0.6°C)	No influence on performance	*T_*gi*_*	CON: 37.0 ± 0.4°C INT: 36.9 ± 0.4°C	CON: 1.6 ± 0.3°C/h INT: 1.8 ± 0.3°C/h (CAL)	CON: 38.9 ± 0.3°C INT: 39.0 ± 0.4°C
Burdon et al., 2013	32°C 40% RH	10	EP: 4 kJ/kg BM cycling time trial	CON: Thermoneutral drink (37°C) INT: Ice slurry (−1°C)	Improved cycle time	*T_*re*_*	CON: 36.9 ± 0.2°C INT: 36.8 ± 0.3°C (NS) (Graph)	CON: 5.3 ± 0.1°C/h INT: 6.2 ± 0.2°C/h (CAL)	CON: 38.7 ± 0.1°C INT: 38.7 ± 0.3°C (NS) (Graph)
Gerrett et al., 2017	31°C 41% RH	12	EPW: 31 min self-paced intermittent running protocol	CON: Water (23°C) INT: Ice slurry (0.1°C)	No influence on distance covered	*T_*gi*_*	CON: 37.2 ± 0.2°C INT: 36.7 ± 0.4°C (S) (Graph)	CON: 3.3 ± 0.2°C/h INT: 3.7 ± 0.3°C/h (CAL)	CON: 38.9 ± 0.3°C INT: 38.6 ± 0.3°C (NS) (Graph)
James et al., 2015	32°C 62% RH	12	EC: Running with increase workload till exhaustion	CON: No cooling INT: Ice slurry (−1°C)		*T_*re*_*	CON: 37.21 ± 0.31°C INT: 36.94 ± 0.31°C (S) (Graph)	CON: 1.11 ± 0.29°C/h INT: 1.38 ± 0.26°C/h (NS)	CON: 39.03 ± 0.45°C INT: 38.96 ± 0.55°C (NS)
Stevens et al., 2016	33°C 46% RH	11	EP: 5 km self-paced running time trial	CON: No cooling INT: Ice slurry (−1°C)	No influence on running time	*T_*re*_*	CON: 37.2 ± 0.4°C INT: 36.9 ± 0.3°C (S)	CON: 4.4 ± 0.2°C/h INT: 4.9 ± 0.2°C/h (CAL)	CON: 39.12 ± 0.25°C INT: 39.04 ± 0.28°C (NS)
Takeshima et al., 2017	30°C 80% RH	10	EC: Cycle at 55% peak power output to exhaustion	CON: No cooling INT: Ice slurry (−1°C)	Longer run time (S)	*T_*re*_*	CON: 37.5 ± 0.3°C INT: 37.1 ± 0.2°C (S)	CON: 2.0 ± 0.2°C/h INT: 2.1 ± 0.2°C/h (CAL)	CON: 39.2 ± 0.3°C INT: 39.2 ± 0.3°C (NS)
Zimmermann and Landers, 2015	33°C 60% RH	9	EPW: 72 min intermittent sprint protocol	CON: Water (25°C) INT: Ice slurry (−0.5°C)	No influence on performance	*T_*gi*_*	CON: 36.7 ± 0.4°C INT: 36.0 ± 0.4°C (S) (Graph)	–	CON: 38.2 ± 0.4°C INT: 37.8 ± 0.4°C (NS) (Graph)
Zimmermann et al., 2017a	35°C 50% RH	10	EPW: 60 min cycling at 55% VO_2peak_	CON: Water INT: Ice slurry	–	*T_*gi*_*	CON: 36.7 ± 0.3°C INT: 36.2 ± 0.1°C (S) (Graph)	CON: 1.3 ± 0.3°C/h INT: 1.5 ± 0.1°C/h (CAL)	CON: 38.0 ± 0.3°C INT: 37.7 ± 0.2°C (S) (Graph)
Zimmermann et al., 2017b	35°C 50% RH	10	EP: 800 kJ cycling time trial	CON: Water INT: Ice slurry	No influence on cycling time	*T_*gi*_*	CON: 37.1 ± 0.4°C INT: 36.4 ± 0.4°C (S)	CON: 1.8 ± 0.3°C/h INT: 2.5 ± 0.2°C/h (CAL)	CON: 39.0 ± 0.5°C INT: 39.0 ± 0.4°C (NS) (Graph)

*RH, relative humidity; EC, exercise capacity; EP, exercise performance; EPW, exercise performance at a fixed workload; S, significant; NS, not significant; CAL, calculated values; REQ, requested values; Graph, graph-extracted values; T_re_, rectal temperature; T_oes_, oesophageal temperature; T_gi_, gastrointestinal temperature; CON, control; INT, intervention*.

**Table 4:** Summary of fluid ingestion studies.

**Study**	**Ambient conditions**	***N* =**	**Exercise protocol**	**Intervention method**	**Exercise outcome**	***T_***c***_* measure**	***T_***c***_* before**	***T_***c***_* rate of rise**	***T_***c***_* end**
**EUHYDRATED STATE WITH LOW FLUID/*****AD LIBITUM*** **vs. HIGH FLUID INTAKE**									
Marino et al., 2004	31.3°C 63.3% RH 2 m/s wind speed	8	EC: Cycle at 70% peak power output to exhaustion	CON: Fluid replacement equal to half the sweat rate INT: Fluid replacement equal to sweat rate	No influence on cycling time	*T_*re*_*	CON: 38.7 ± 0.4°C INT: 38.6 ± 0.5°C (REQ)	–	CON: 39.0 ± 0.4°C INT: 38.8 ± 0.6°C (NS)
Dugas et al., 2009	33°C 50% RH	6	EP: 80 km cycling time trial	CON: Fluid ingested to replace 33% of weight lost INT: Fluid ingested to replace 100% of weight lost	No influence on cycling time	*T_*re*_*	CON: 36.8 ± 0.1°C INT: 36.9 ± 0.2°C (NS)	–	CON: 39.2 ± 0.5°C INT: 38.9 ± 0.4°C (NS)
Montain and Coyle, 1992a	33°C 50% RH 2.5 m/s wind speed	8	EPW: 2 h cycle at a power output equal to 62–67% maximal oxygen consumption	CON: Small (50%) fluid replacement INT: Large (80%) fluid replacement	–	*T_*oes*_*	CON: 37.01 ± 0.20°C INT: 37.01 ± 0.26°C (REQ)	CON: 0.60 ± 0.14°C/h INT: 0.47 ± 0.18°C/h (REQ)	–
McConell et al., 1997	21°C 43% RH	7	EPW: 2 h cycle at 60% VO_2_ peak	CON: 50% fluid replacement INT: 100% fluid replacement	–	*T_*re*_*	CON: 37.2 ± 0.2°C INT: 37.1 ± 0.2°C (REQ)	CON: 0.8 ± 0.3°C/h INT: 0.7 ± 0.1°C/h (REQ)	–
Bardis et al., 2017	AD: 31.4 ± 0.5°C PD: 31.7 ± 0.4°C (NS) 6.4 m/s	10	EPW: 3 sets of 5 km cycling at 50% maximal power output followed by 5 km cycling all out at 3% grade (Total 30 km)	CON: *ad libitum* water intake INT: Fluid ingested to replace 100% of fluid lost via sweating	Faster cycling speed (S)	*T_*gi*_*	CON: 37.4 ± 0.1°C INT: 37.6 ± 0.2°C (NS) (Graph)	–	CON: 38.7 ± 0.4°C INT: 38.4 ± 0.4°C (S) (Graph)
James L. J. et al., 2017	34°C 50% RH 0.3–0.4 m/s	7	EPW: 15 min cycling performance test	CON: Fluid replacement to induce 2.5% body mass loss INT: Fluid replacement to replace sweat loss	More work completed (S)	*T_*gi*_*	CON: 37.0 ± 0.2°C INT: 37.2 ± 0.3°C (Graph)	CON: 6.8 ± 1.8°C/h INT: 4.4 ± 2.3°C/h (CAL)	CON: 38.7 ± 0.5°C INT: 38.3 ± 0.5°C
Périard et al., 2014	37°C 33% RH	10	EPW: 20 min tennis match	CON: *ad libitum* water intake INT: Fluid ingested to match 70% of sweat loss	–		CON: 37.8 ± 0.3°C INT: 37.7 ± 0.3°C (NS) (Graph)	CON: 4.8 ± 1.75°C/hINT: 4.5 ± 2.0°C/h(CAL)	CON: 39.4 ± 0.5°C INT: 39.2 ± 0.6°C (NS)
**EUHYDRATED STATE WITH NO FLUID VS. HIGH FLUID INTAKE**									
Marino et al., 2004	31.3°C 63.3% RH 2 m/s wind speed	8	EC: Cycle at 70% peak power output to exhaustion	CON: No fluid replacement INT: Fluid replacement equal to sweat rate	Longer time to exhaustion (S)	*T_*re*_*	CON: 38.8 ± 0.4°C INT: 38.6 ± 0.5°C (NS)	–	CON: 39.2 ± 0.4°C INT: 38.8 ± 0.6°C (NS)
Hargreaves et al., 1996	20–22°C	5	EPW: 2 h cycle at 67% VO_2_ peak	CON: No fluid ingested INT: Ingestion of fluid to prevent loss of body mass	–	*T_*re*_*	CON: 36.7 ± 0.2°C INT: 36.7 ± 0.4°C (NS)	CON: 0.9 ± 0.3°C/h INT: 0.6 ± 0.3°C/h (CAL)	–
Armstrong et al., 1997	33°C 56% RH 0.1 m/s air speed	10	EPW: 90 min treadmill walk at 5.6 km/h, 5% grade	CON: No water intake INT: *ad libitum* water intake	–	*T_*re*_*	–	CON: 0.7 ± 0.2°C/h INT: 0.6 ± 0.2°C/h (CAL)	–
Robinson et al., 1995	20°C 60% RH 3 m/s air speed	8	EP: 60 min cycle to achieve greatest possible distance	CON: No fluid ingested INT: Ingestion of fluid to replace approximate sweat loss	Less distance covered (S)	*T_*re*_*	CON: 36.8 ± 0.3°C INT: 36.5 ± 0.6°C (NS)	–	CON: 38.6 ± 0.6°C INT: 38.1 ± 0.6°C (NS)
Fallowfield et al., 1996	20°C	8	EC: Treadmill run at 70% VO_2_ max to exhaustion	CON: No fluid ingested INT: Fluid replacement before and during exercise	Longer time to exhaustion (S)	*T_*re*_*	–	–	CON: 38.8 ± 1.1°C INT: 39.1 ± 0.6°C (NS)
Coso et al., 2008	36°C 29% RH 1.9 m/s airflow	7	EPW: 120 min cycle at 63% VO_2_ max	CON: No fluid ingested INT: Ingestion of mineral water	–	*T_*re*_*	CON: 37.6 ± 0.3°C INT: 37.6 ± 0.3°C (NS)	CON: 0.9 ± 0.2°C/h INT: 0.6 ± 0.2°C/h (CAL)	–
Cheung and McLellan, 1997	40°C 30% RH	8	EC: Either a light (3.5 km/h, 0% grade) or a heavy (4.8 km/h, 4% grade) treadmill walk to exhaustion	CON: No fluid replacement INT: Fluid replacement	Longer time to exhaustion (S) for light exercise	*T_*re*_*	CON_light_: 36.89 ± 0.29°C INT_light_: 36.85 ± 0.28°C (NS) CON_heavy_: 36.88 ± 0.21°C INT_heavy_: 36.94 ± 0.27°C (NS)	CON_light_: 1.19 ± 0.46°C/h INT_light_: 1.15 ± 0.32°C/h (CAL) CON_heavy_: 1.88 ± 0.32°C/h INT_heavy_: 1.76 ± 0.42°C/h (CAL)	CON_light_: 38.74 ± 0.68°C INT_light_: 38.90 ± 0.40°C (NS) CON_heavy_: 38.71 ± 0.43°C INT_heavy_: 38.69 ± 0.62°C (NS)
Munoz et al., 2012	33°C 30% RH	10	EP: 5 km running time trial	CON: No rehydration INT: Oral rehydration	No influence on performance time	*T_*re*_*	CON: 37.78 ± 0.41°C INT: 37.57 ± 0.31°C (NS)	–	CON: 39.19 ± 0.45°C INT: 38.97 ± 0.36°C (NS)
Kay and Marino, 2003	33.2°C 63.3% RH	7	EP: 60 min cycle to achieve greatest possible distance	CON: No fluid ingested INT: Fluid ingested to prevent any change in body mass	No influence on distance cycled	*T_*re*_*	–	–	CON: 38.9 ± 0.5°C INT: 38.7 ± 0.4°C (NS)
Dugas et al., 2009	33°C 50% RH	6	EP: 80 km cycling time trial	CON: No fluid ingested INT: Fluid ingested to replace 100% of weight lost	No influence on cycling time	*T_*re*_*	CON: 36.8 ± 0.2°C INT: 36.9 ± 0.2°C (NS)	–	CON: 39.2 ± 0.4°C INT: 38.9 ± 0.4°C (NS)
Hasegawa et al., 2006	32°C 80% RH	9	EPW: 60 min cycle at 60% VO_2_ max	CON: No water intake INT: Water ingestion at 5 min intervals	–	*T_*re*_*	CON: 37.37 ± 0.15°C INT: 37.37 ± 0.16°C (REQ)	CON: 1.77 ± 0.22°C/h INT: 1.39 ± 0.27°C/h (REQ)	–
Gagnon et al., 2012	42°C 20% RH 1 m/s air speed	8 untrained 8 trained	EPW: 120 min cycle at 120 W	CON: No fluid replacement INT: Fluid replacement	–	*T_*oes*_*	CON_UT_: 37.23 ± 0.57°C INT_UT_: 36.96 ± 0.25°C CON_T_: 36.80 ± 0.28°C INT_T_: 36.69 ± 0.25°C (NS)	CON_UT_: 0.74 ± 0.28°C/h INT_UT_: 0.70 ± 0.18°C/h CON_T_: 1.20 ± 0.25°C/h INT_T_: 0.81 ± 0.24°C/h (CAL)	–
Montain and Coyle, 1992b	33°C 50% RH 2.5 m/s wind speed	8	EPW: 2 h cycle at a power output equal to 62–67% maximal oxygen consumption	CON: No fluid replacement INT: Large (80%) fluid replacement	–	*T_*oes*_*	CON: 36.99 ± 0.36°C INT: 37.01 ± 0.26°C (REQ)	CON: 0.84 ± 0.24°C/h INT: 0.47 ± 0.18°C/h (REQ)	–
McConell et al., 1997	21°C 43% RH	7	EPW: 2 h cycle at 60% VO_2_ peak	CON: No fluid replacement INT: 100% fluid replacement	–	*T_*re*_*	CON: 37.1 ± 0.2°C INT: 37.1 ± 0.2°C (REQ)	CON: 1.0 ± 0.2°C/h INT: 0.7 ± 0.1°C/h (REQ)	–
Wall et al., 2015	33°C 40% RH 32 km/h	10	EPW: 25 km cycling time trial	CON: No fluid replacement INT: 100% fluid replacement	No influence on cycling time	*T_*re*_*	CON: 37.1 ± 0.2°C INT: 37.0 ± 0.2°C (NS) (Graph)	CON: 2.6 ± 0.5°C/h INT: 2.49 ± 0.53°C/h (CAL)	CON: 38.9 ± 0.3°C INT: 38.7 ± 0.3°C (S) (Graph)
Wittbrodt et al., 2015	32°C 65% RH	12	EPW: 50 min cycling at 60% VO_2peak_	CON: No fluid intake INT: 100% fluid replacement	–	*T_*re*_*	CON: 37.0 ± 0.3°C INT: 36.8 ± 0.8°C (NS) (Graph)	CON: 1.4 ± 0.7°C/h INT: 1.0 ± 1.3°C/h (CAL)	CON: 38.2 ± 0.5°C INT: 37.6 ± 0.7°C (S) (Graph)
Trangmar et al., 2015	35% 50% RH	8	EC: Cycling at 60% VO_2max_ until volitional exhaustion	CON: No fluid intake INT: Fluid intake to replace body mass loss	Shorter exercise duration (S)	*T_*gi*_*	CON: 37.4 ± 0.1°C INT: 37.3 ± 0.1°C (NS)	–	CON: 38.7 ± 0.1°C INT: 38.2 ± 0.2°C (S)
**HYPOHYDRATED STATE WITH NO FLUID vs. HIGH FLUID INTAKE**									
Armstrong et al., 1997	33°C 56% RH 0.1 m/s air speed	10	EPW: 90 min treadmill walk at 5.6 km/h, 5% grade	CON: No water Intake INT: *ad libitum* water intake	–	*T_*re*_*	–	CON: 1.2 ± 0.2°C/h INT: 0.7 ± 0.2°C/h (CAL)	–

*RH, relative humidity; EC, exercise capacity; EP, exercise performance; EPW, exercise performance at a fixed workload; S, significant; NS, not significant; CAL, calculated values; REQ, requested values; Graph, graph-extracted values; T_re_, rectal temperature; T_oes_, oesophageal temperature; T_gi_, gastrointestinal temperature; CON, control; INT, intervention*.

### Effect of Heat Mitigation Strategies on T_c_

**Figure 2:**
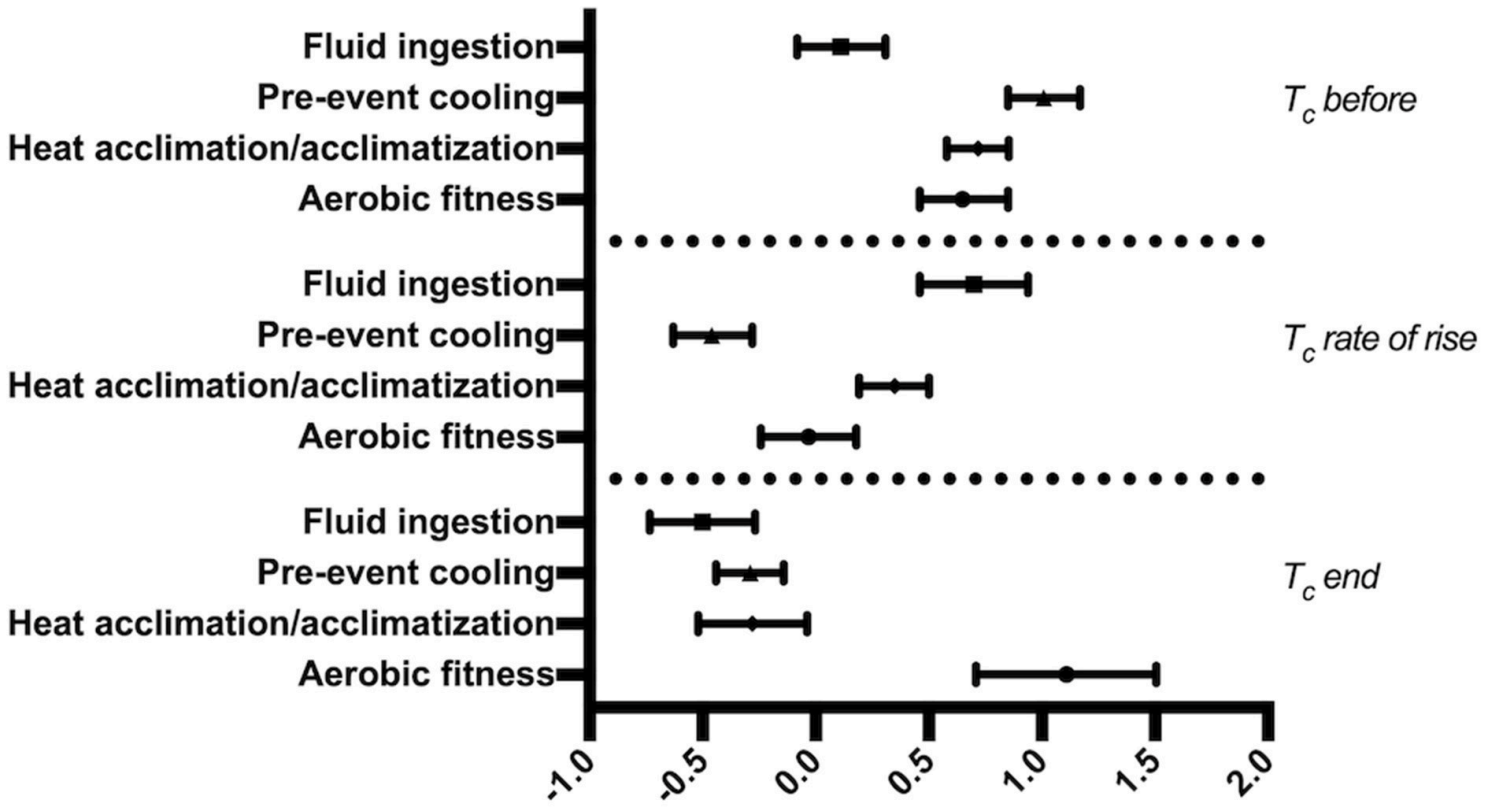
Forest plot of Hedges' g weighted averages of heat mitigation strategies effect on T_c_ at different points.

PC was found to be the most effective in the lowering of T_c_ before exercise (Hedge's g = 1.01; 95% Confidence Intervals 0.85–1.17; Figure 2). A moderate effect on lowering of T_c_ before exercise was observed for HA (0.72; 0.58 to 0.86) and AF (0.65; 0.46 to 0.85) while FI (0.11; −0.08 to 0.31) only exhibited a trivial effect on lowering T_c_ before exercise.

Rate of rise of T_c_ during exercise was most attenuated by FI (0.70; 0.46 to 0.94), followed by HA (0.35; 0.19 to 0.50). AF (−0.03; −0.24 to 0.18) showed a trivial effect on the rate of rise of T_c_ while PC (−0.46; −0.63 to −0.28) did not appear to be as effective in lowering the rate of rise of T_c_.

AF (1.11; 0.71 to 1.51) exhibited a large effect on extending the limit of T_c_ at the end of exercise. However, HA (−0.28; −0.52 to −0.04), PC (−0.29; −0.44 to −0.14), and FI (−0.50; −0.74 to −0.27) did not seem as effective in extending the T_c_ limit at the end of exercise.

**Figure 3:**
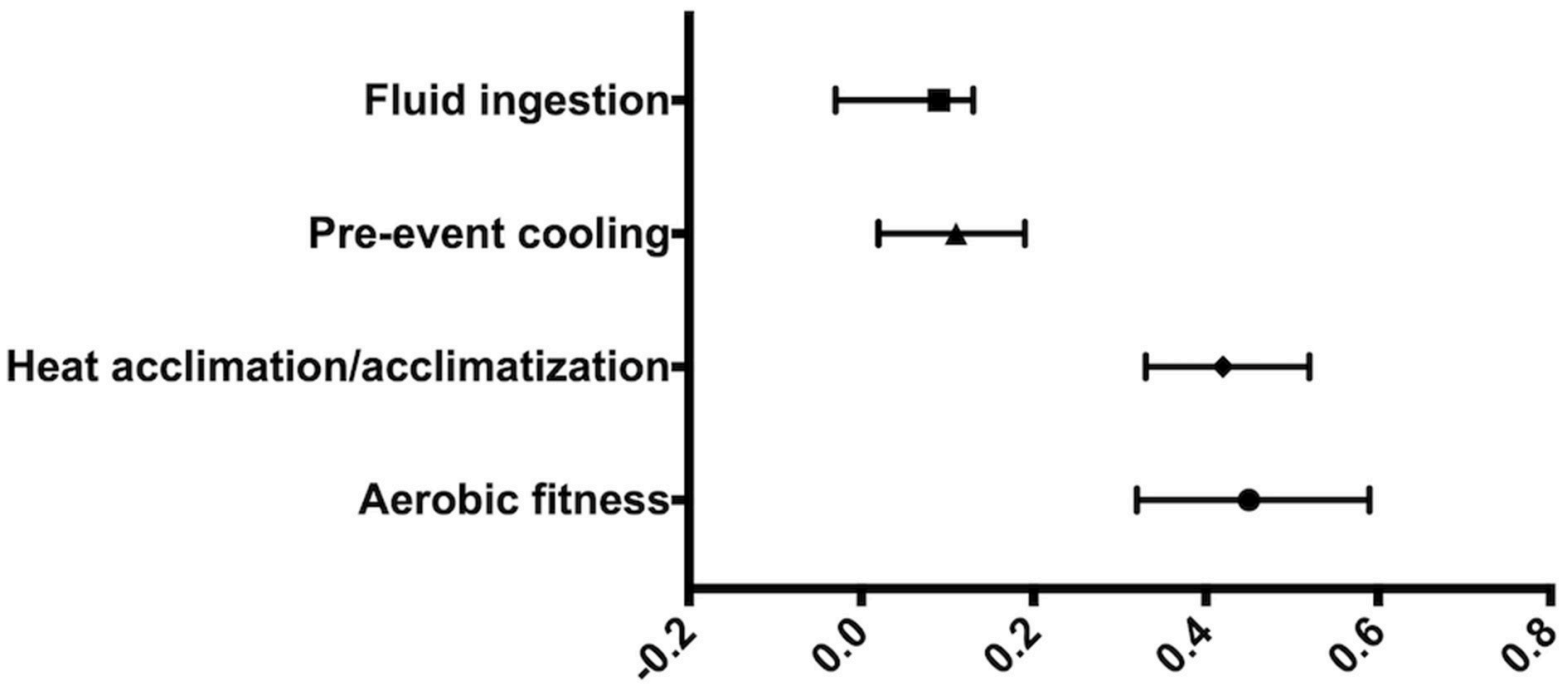
Forest plot of combined Hedges' g weighted averages of heat mitigation strategies.

In combination, AF was found to be the most effective at favorably altering T_c_ (0.45; 0.32 to 0.59), followed by HA (0.42; 0.33 to 0.52), PC (0.11; 0.02 to 0.19) and FI (0.09; −0.03 to 0.13) (Figure 3).

In addition, AF studies included both longitudinal and cross-sectional studies. We sought to determine if there was an effect on T_c_ variables when comparing “between subjects” and “within subjects” studies. We found that effect sizes were comparable with “between subjects” AF studies (0.45; 0.28 to 0.61) and “within subjects” AF studies (0.38; 0.14 to 0.61). The large overlap in CIs suggest that the inclusion of both study types did not have significantly different effects on T_c_ variables.

### Effect of Heat Mitigation Strategies on Endurance

Of the 118 articles selected and used for analysis of the strategies based on effects on T_c_, 45 studies also included measurements of endurance. The number of studies for each heat mitigation strategy is as follows: AF (*n* = 5), HA (*n* = 7), PC (*n* = 24), and FI (*n* = 9).

**Figure 4:**
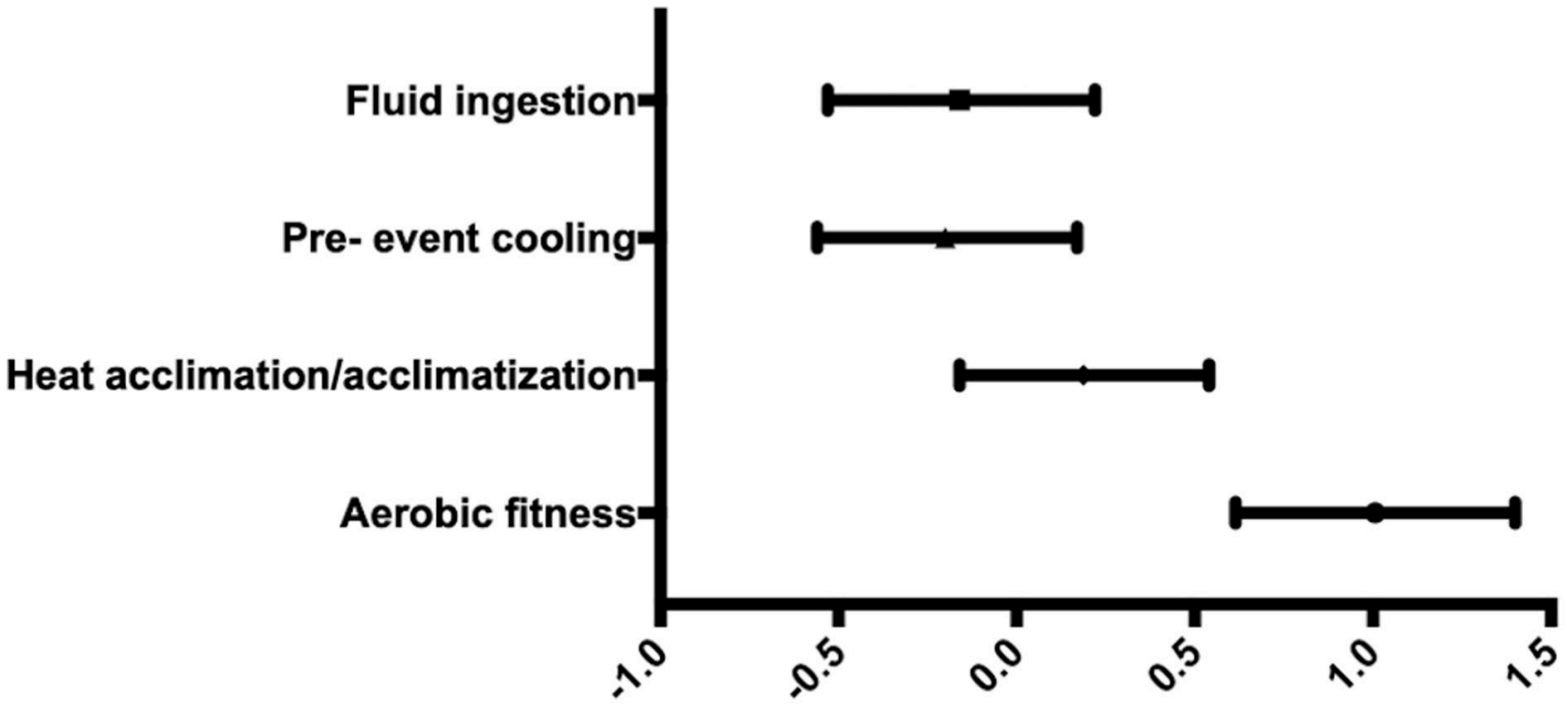
Forest plot of Hedge's g weighted averages of heat mitigation strategies on endurance.

We observed that AF was the most effective in improving endurance (1.01; 1.40 to 0.61), followed by HA (0.19; −0.16 to 0.54), FI (−0.16; −0.53 to 0.22), and PC (−0.20; −0.56 to 0.17) (Figure 4).

## Discussion

This meta-analysis aimed to evaluate the efficacy of different heat mitigation strategies. Our main findings suggest that AF was most effective in altering T_c_, followed by HA, PC and FI. A secondary objective was to evaluate the effect of these strategies on endurance. We observed that aerobic fitness was again the most beneficial, followed by heat acclimation/acclimatization, fluid ingestion and pre-cooling. It is noteworthy that the ranking of the effectiveness of the heat mitigation strategies on favorably altering T_c_ is similar to their effectiveness in improving endurance (Table 5).

**Table 5:** Ranking of heat mitigation strategies based on Hedges' g weighted averages.

	**Combined Hedge's g weighted averages effect on T_**c**_**	**Rank**	**Combined Hedge's g weighted averages effect on performance and/or capacity**	**Rank**
Aerobic Fitness	0.45	1	1.01	1
Heat acclimation/acclimatization	0.42	2	0.19	2
Pre-exercise cooling	0.11	3	−0.20	4
Fluid ingestion	0.09	4	−0.16	3

### Aerobic Fitness

Individuals with a higher aerobic fitness have been shown to have a lower pre-exercise T_c_ at rest (Selkirk and McLellan, 2001; Mora-Rodriguez et al., 2010). Aerobic fitness also enhances heat dissipation by lowering the threshold T_c_ at which both skin vasodilation and sweating occur (Nadel et al., 1974; Ichinose et al., 2009). Kuwahara et al. (2005) found that sweat rates of trained individuals were significantly higher than that of untrained individuals over a 30 min cycling exercise and that the onset of sweating occurred earlier on in the exercise as well. Higher aerobic fitness has also shown to cause an increase in skin blood flow (Fritzsche and Coyle, 2000). The combination of these two effects will lower T_c_ by enhancing heat dissipation during exercise in the heat. In addition, a greater aerobic fitness elicits a higher T_c_ attained at the end of exercise (Cheung and McLellan, 1998b; Selkirk and McLellan, 2001). This is corroborated by studies in marathon runners, where highly aerobically trained individuals were able to tolerate greater end T_c_ without any pathophysiological effects (Maron et al., 1977; Byrne et al., 2006). However, it should be noted that the ability to extend the limit of T_c_ at the end of exercise may pose as a double-edged sword, as highly motivated individuals may continue to exercise past the limits of acceptable T_c_ which could cause higher rates of exertional heat related illnesses occurring.

### Heat Acclimation/Acclimatization

Heat acclimation/acclimatization refers to the physiological adaptations that occur as a result of prolonged, repeated exposure to heat stress (Armstrong and Maresh, 1991). It is noteworthy that the magnitude and duration of the heat acclimation/acclimatization protocols are important considerations in the development of the above physiological adaptations (Tyler et al., 2016). Previous meta-analysis and studies have shown that effects on cardiovascular efficiency and T_c_ may be achieved in protocols lasting less than 7 days, while thermoregulatory adaptations and improvements in endurance capacity and performance may require up to 14 days. For the benefits to be maximized, protocols longer than 2 weeks may also be considered (Armstrong and Maresh, 1991; Pandolf, 1998; Tyler et al., 2016). Heat acclimation/acclimatization has been shown to effectively reduce pre-exercise body temperature (Nielsen et al., 1993; Cotter et al., 1997). The physiological adaptations also observed include decreased heart rate (Harrison, 1985; Lorenzo and Minson, 2010), increased cardiac output (Harrison, 1985; Nielsen, 1996) and plasma volume (Mitchell et al., 1976; Lorenzo and Minson, 2010). Most significantly, cutaneous vasodilation occurs at a lower T_c_ threshold, together with an increase in skin blood flow (Roberts et al., 1977). The onset of sweating also occurs at a lower T_c_ threshold, resulting in increased sweat rates during exercise (Cotter et al., 1997; Cheung and McLellan, 1998a). Taken together, this helps to reduce the rate of rise of T_c_ during exercise due to increased cardiovascular efficiency and heat dissipation mechanisms.

However, for tropical natives, heat acclimatization does not lead to more efficient thermoregulation. In a study by Lee and colleagues (Lee et al., 2012), military soldiers native to a warm and humid climate were asked to undergo a 10 day heat acclimatization programme. Although there was an increase in work tolerance following acclimatization, no significant cardiovascular or thermoregulatory adaptations were found. These observations could suggest that thermoregulatory benefits of heat acclimatization are minimized in tropical natives, possibly due to the “partially acquired heat acclimatization status from living and training in a warm and humid climate” (Lee et al., 2012). Alternatively, thermoregulatory benefits from heat acclimatization may also be minimized in tropical natives due to modern behavioral adaptations such as the usage of air conditioning in living spaces and the avoidance of exercise during the hottest periods of the day that reduce the environmental heat stimulus experienced (Bain and Jay, 2011). In addition, evaporative heat loss through sweating is compromised with high relative humidity and therefore results in a higher rate of rise of T_c_ during exercise (Maughan et al., 2012).

It is also noteworthy that heat acclimation/acclimatization encompasses aerobic fitness as well. In most protocols, there is some form of training in the simulated laboratory settings or in the natural environmental settings. Few studies have attempted to separate the effects of heat acclimation from aerobic fitness. A study by Ravanelli et al. (2018) showed that a greater maximum skin wittedness occurred at the end of aerobic training in temperate conditions (22°C, 30% relative humidity), and this was further augmented by heat acclimation in a hot and humid condition (38°C, 65% relative humidity). This suggests that studies that include aerobic training in the heat acclimation/acclimatization protocols may have had their thermoregulatory effects augmented. However, as there have been few studies that have isolated the effects of heat acclimation/acclimatization from aerobic training or compared exertional vs. passive exposure to heat in heat acclimation/acclimatization protocols, it would be difficult to isolate the effects of heat acclimation/acclimatization from aerobic fitness.

### Pre-exercise Cooling

The main intention of pre-exercise cooling is to lower T_c_ before exercise to extend heat storage capacity in hope to delay the onset of fatigue and in this review, we have observed pre-exercise cooling to be most effective in this aspect compared to the other heat mitigation strategies. For comprehensive reviews on pre-exercise cooling (see Marino, 2002; Quod et al., 2006; Duffield, 2008; Jones et al., 2012; Siegel and Laursen, 2012; Wegmann et al., 2012; Ross et al., 2013). The various pre-exercise cooling methods include cold water immersion (Booth et al., 1997; Kay et al., 1999), cold air exposure (Lee and Haymes, 1995; Cotter et al., 2001), cold vest (Arngrimsson et al., 2004; Bogerd et al., 2010), cold fluid ingestion (Lee et al., 2008; Byrne et al., 2011), and ice slurry ingestion (Siegel et al., 2010; Yeo et al., 2012).

Largely, the methods above have been shown to be effective in lowering T_c_ pre-exercise, which could consequently reduce thermal strain and therefore enhance endurance performance. Apart from lowering T_c_ pre-exercise, ice slurry ingestion has shown to increase T_c_ at the end of exercise. In both laboratory and field studies, T_c_ was higher at the end of exercise with ice slurry. In the laboratory study by Siegel et al. (2010) oesophageal temperature was higher by 0.31°C, and in the field study by Yeo et al. (2012), gastrointestinal temperature was higher by 0.4°C with the ingestion of ice slurry. Siegel et al. (2010) suggested that the ingestion of ice slurry may have affected thermoreceptors present causing a “physiologically meaningful reduction in brain temperature.” In addition, ice slurry ingestion may have potentially attenuated any afferent feedback that would have resulted in central reduction in muscle activation, allowing tolerance of a greater thermoregulatory load (Lee et al., 2010).

In addition, practitioners should consider the magnitude of pre-exercise cooling strategies being employed. Large volumes of ice slurry/cold water ingestion may blunt heat loss pathways by limiting sweat gland activity. This would reduce evaporative heat loss which may counteract to cause a greater heat storage and higher T_c_ during exercise which would be unfavorable (Ruddock et al., 2017). However, it should be noted that this potentially negative effect of ice slurry/cold water ingestion may be a greater concern in dry environments as compared to humid environments. In hot and humid environments, despite reductions in evaporative heat loss potential, actual evaporation may not be reduced, and ice slurry/cold water ingestion would still be beneficial in reducing body heat storage. This is due to the attainment of the maximum evaporation potential anyway, and any additional sweat generated would drip off the skin in hot and humid environments (Jay and Morris, 2018). Numerous studies also support the effectiveness of pre-exercise ice slurry/cold water ingestion in lowering T_c_ and demonstrate that this profile is continued during exercise (Lee et al., 2008; Siegel et al., 2010, 2012; Byrne et al., 2011; Yeo et al., 2012).

The effectiveness of pre-cooling as a strategy in altering T_c_ may be limited as it is mostly done acutely before exercise. As such, its benefit may not be able to be sustained throughout the exercise duration. To counteract this limitation, considerations can be made to consider per/mid-exercise cooling. Whilst not discussed in the present meta-analysis, previous reviews have shown that per/mid-exercise cooling may be as effective in enhancing exercise performance in hot environments (Bongers et al., 2015, 2017).

### Fluid Ingestion

Fluid ingestion is a common strategy used to reduce thermoregulatory strain in the heat. Many studies have shown that when fluid is ingested during exercise, exercise capacity and performance are enhanced (Fallowfield et al., 1996; Cheung and McLellan, 1997; Marino et al., 2004). A more controversial issue is the optimal amount of fluid to be consumed during exercise. Two dominant viewpoints exist—the first is that athletes should prevent fluid loss of >2% body mass (Sawka et al., 1985; Montain and Coyle, 1992a; Sawka and Coyle, 1999; Casa et al., 2010), while the other recommends drinking *ad libitum* (Noakes, 1995; Beltrami et al., 2008; Lee et al., 2011) due to an increased prevalence of exercise associated hyponatremia, commonly referred to as water intoxication (Noakes, 1995). Even in warm conditions where sweat rates are high, the behavioral drive to ingest fluids could exceed the physiological sweat loss (Lee et al., 2011).

This review analyzed the effects of a (i) low fluid/*ad libitum* vs. high fluid intake and (ii) no fluid vs. high fluid intake on T_c_. All participants began exercise in a euhydrated state. Dugas et al. (2009) found that *ad libitum* drinking while cycling replaces approximately 55% of fluid losses., while Daries et al. (2000) found that *ad libitum* drinking during a treadmill run replaces approximately 30% of fluid losses. Hence in this evaluation, a fluid intake trial replacing closest to ~45% of fluid losses was chosen to represent the low fluid/*ad libitum* condition. It should also be stated that the results in trials in which the control state was no fluid intake may have exaggerated the results of fluid ingestion seen in this meta-analysis. This is especially so when we consider that it is impractical during a competition event to avoid drinking. As such, future hydration studies should consider avoiding a “No fluid” control state.

Ideally, individuals should begin their exercise in a euhydrated state. This could be achieved by drinking 6 mL of water per kg body mass for 2–3 h pre-exercising in a hot environment (Racinais et al., 2015a). During exercise, fluid is largely loss through sweating. Sweat rates may vary depending on individual characteristics, environmental conditions and heat acclimation/acclimatization status (Cheuvront et al., 2007). Practitioners should therefore consider determining their sweat rate prior to exercising in a hot environment to determine the amount of rehydration or fluid intake that is necessary to reduce physiological strain and optimize performance, without increasing body weight. Considerations can also be made to include supplementation with sodium (Casa, 1999; Sawka et al., 2007) and glucose (von Duvillard et al., 2007; Burke et al., 2011).

## Practical Implications

Logically, employing a combination of all the different heat mitigation strategies would be most beneficial in extending an athlete's heat storage capacity and in optimizing exercise performance in the heat. However, due to time and resource constraints, it may not be practical for athletes and coaches to employ all these strategies for competition. By knowing which heat mitigation strategy is most effective, an informed decision can be made. Strategies such as aerobic fitness and heat acclimation/acclimatization have to be conducted months and weeks respectively before competition in order to reap its benefits. On the other hand, strategies such as pre-exercise cooling and fluid ingestion can be done immediately before or during competition. Practicality and comfort should be the main focus when deciding which heat mitigation strategy to employ. For example, pre-exercise cooling methods such as cold water immersion may be effective in lowering T_c_ before exercise begins. However, it may be cumbersome to set up a cold water bath especially during outdoor field events. Furthermore, being immersed in a cold water bath may be an uncomfortable experience for some athletes, and may cool the muscles prior to the event and hence is not practical to be used prior to competition (Quod et al., 2006; Ross et al., 2013). It is noteworthy that there could be inter-individual differences when employing each of these heat mitigation strategies. Athletes and coaches are advised to experiment with these strategies during training before deciding on the appropriate strategy to employ during competition. Finally, the importance of the usage of heat mitigation strategies when competing in hot and humid environments cannot be stressed enough. From this meta-analysis, we have shown that aerobic fitness is the most effective heat mitigation strategy. However, this does not understate the importance of a combination of heat mitigation strategies, nor does it reflect that should an athlete be aerobically fit, other heat mitigation strategies are not necessary. In the 15th International Association of Athletics Federations (IAAF) World Championships held in Beijing (China), mean and maximal temperatures were anticipated to be 26° and 33°C respectively, with relative humidity of ~73%. Despite the expected hot and humid conditions, only 15% of athletes reported having specifically prepared for these conditions. Of these, females and athletes with previous history of exertional heat illnesses (EHI) were more likely to adopt heat mitigation strategies (Périard et al., 2017). Although <2% experienced EHI symptoms, athletes should be more aware of the potential benefits of using one or more heat mitigation strategies in the lead up to competitions in hot and humid environments. As global temperatures continue to rise, the importance of such heat mitigation strategies in enhancing performance and in reducing the likelihood of EHI cannot be understated.

## Limitations

The methodology of using a meta-analysis to evaluate effectiveness of different strategies is not without limitation. Publication and language restriction bias may have affected the number of studies that could be included in the analysis. As such, care was taken to ensure to control for such biases, such as a manual tracking of review articles to ensure that studies that were relevant but that did not show up in the initial search of the databases could be included as well. The heterogeneity of the included studies was also controlled for by statistical analysis. In addition, due to the practical difficulty in blinding the participants to the heat mitigation strategy being employed, any beneficial effect arising from the placebo effect could not be eliminated.

This meta-analysis also did not include behavioral alterations that could be undertaken as a mitigation strategy against exertional heat stress. Taking regular breaks during exercise is an effective way to minimize heat strain by preventing an excessive rise of T_c_ and increasing exercise tolerance in the heat (Minett et al., 2011). Individuals should also avoid exercising during the hottest part of the day. Alternatively, several shorter sessions of exercise can be performed rather than having a single long session, to reduce hyperthermia, while maintaining the quality of the exercise session (Maughan and Shirreffs, 2004). When exercising in the heat, an important consideration is to ensure that the material in the clothing does not prevent the evaporation of sweat from the skin (Maughan and Shirreffs, 2004). Furthermore, black and dark-colored clothing absorb more heat and should not be worn when exercising in the heat. For a review of the thermal characteristics of clothing (see Gonzalez, 1988; Parsons, 2002). One reason for the exclusion is that there is often time pressure to complete a task or race as fast as possible and/or in certain attire that does not permit behavioral alteration during competitions. There are also few studies that looked at the effect of behavioral alterations on endurance that fulfilled our inclusion criteria, which did not allow for the calculation of an effect size to compare effectively with the other heat mitigations strategies.

Although these limitations should be accounted for, this is the first meta-analysis to compare several different heat mitigation strategies and their effects on T_c_ and endurance. As such, this meta-analysis could provide the information necessary to allow for more informed decision making by coaches, athletes and sports scientists during exercise in hot and/or humid environments.

## Conclusion

In conclusion, aerobic fitness was found to be the most effective heat mitigation strategy, followed by heat acclimation/acclimatization, pre-exercise cooling and lastly, fluid ingestion. The similarity in ranking between the ability of each heat mitigation strategy to favorably alter T_c_ and affect endurance suggest that alteration of heat strain may be a key limiting factor that contributes to endurance. This analysis has practical implications for an athlete preparing for competition in the heat and also allows coaches and sport scientists to make a well-informed and objective decision when choosing which heat mitigation strategy to employ.

## Author Contributions

SA and PT realized the research literature. SA, PT, and JL contributed to the writing of the manuscript.

### Conflict of Interest Statement

The authors declare that the research was conducted in the absence of any commercial or financial relationships that could be construed as a potential conflict of interest.
